# Natron glass beads reveal proto-Silk Road between the Mediterranean and China in the 1st millennium BCE

**DOI:** 10.1038/s41598-021-82245-w

**Published:** 2021-02-11

**Authors:** Qin-Qin Lü, Julian Henderson, Yongqiang Wang, Binghua Wang

**Affiliations:** 1 Department for the History of Science and Scientific Archaeology, University of Science and Technology of China, Hefei, 230026 China; 2 Department of Classics and Archaeology, University of Nottingham, University Park, Nottingham, NG7 2RD UK; 3 University of Nottingham, Ningbo, 315100 China; 4 Xinjiang Institute of Cultural Relics and Archaeology, Urumqi, 830001 China

**Keywords:** Mineralogy, Materials science

## Abstract

Natron-based glass was a vital part of material culture in the Mediterranean and Europe for nearly two millennia, but natron glass found elsewhere on the Eurasian Continent has not received adequate discussion, despite its influence on ancient Asian glass. Here we present a new interpretation of natron glass finds from both the West and the East. After establishing the compositional types and technological sequence of Mediterranean natron glass (eighth-second century BCE) using trace elements, we report the analysis of a mid-1st millennium BCE glass bead from Xinjiang, China, which was likely made with Levantine raw glass, and identify common types of stratified eye beads in Eurasia based on a compositional and typological comparison. Combining these findings, we propose that a considerable number of Mediterranean natron glass products had arrived in East Asia at least by the fifth century BCE, which may have been a contributing factor in the development of native Chinese glass-making. The swift diffusion of natron glass across Eurasia in the 1st millennium BCE was likely facilitated by a three-stage process involving maritime and overland networks and multiple forms of trade and exchange, indicating a highly adaptable and increasingly efficient transcontinental connection along the ‘Proto-Silk Road’.

## Introduction

Past inter-regional interactions in the Eurasian Continent shaped our world in numerous ways. It is critically important to understand how such interactions evolved in time, extended to larger areas, and exerted increasing influence over cultures. Recent studies have revealed prehistoric connections in Eurasia based on evidence such as human migration^1–3^, the transmission of domesticated crops^4,5^, and the diffusion of pottery^6^ and metallurgical^7^ technologies. However, before and during much of the Bronze Age, the spread of population, materials, and innovations remained generally time-consuming, suggesting limited intensity of long-range communications. For instance, wheat did not reach the lower Yellow River in China until six millennia after its domestication in West Asia^8,9^. This situation had changed by the second century BCE, when Chinese Warring States silk, mirrors and lacquer appeared in contemporaneous burials in Siberia and Inner Asia^10^, and remote regions were recorded in Roman and Chinese literature. At the dawn of a surge in transcontinental material exchange, the Han Dynasty’s envoy Zhang Qian was sent for missions into Asia’s heartland in the late second century BCE, an event conventionally considered as the beginning of the historical Silk Road. The preceding period, i.e., the 1st millennium BCE, is therefore vital for the decisive acceleration and integration of the long-distance interactions in Eurasia.

Man-made glass is one of the truly transnational products of civilization, and can be used to investigate long-distance interactions. Glass had a diverse history of origin and dispersal. Soda glass was first manufactured in the Near East, and subsequently in Europe, Central Asia, and South Asia. Potash glass can be found in South, Southeast, and East Asia, as well as in Europe. Lead-barium glass first appeared in China and was also found in other parts of East Asia^11,12^. The emergence of glass-making technology in East Asia during the 1st millennium BCE is an open question. Archaeological investigations of early Chinese glass have revealed the presence of all three major groups^13–15^, suggesting a period active in cultural interaction and technological exploration. Provenancing these early artifacts is essential for determining the origin of Chinese and Asian glass.

Natron-based glass is a type of soda glass made using the natron mineral as the flux, characterized by low magnesia and low potash contents^16^. Natron glass appeared in the early 1st millennium BCE, and was prevalent to the west of the Euphrates until about 800 CE. Natron glass is well suited for studying transcontinental connection in the 1st millennium BCE since it was only produced in certain areas in the Mediterranean. In recent decades, our understanding of natron glass has accumulated significantly, especially for those of and after the Roman Empire. However, for natron glass in the 1st millennium BCE such as Early Iron Age glass, Phoenician glass, and Hellenistic glass, questions about their raw materials, production activities, and dispersion routes still remain. Moreover, for eastern Eurasian, very few natron glass artifacts dated to before the Han Dynasty (206 BCE–220 CE) have been reported. However, if the earliest Chinese glasses were imported from the West, a large portion of those should have been natron glass, which at the time had become dominant in the West. Indeed, the history of the eastward dispersion of natron glass is not well known: we will attempt to redress this imbalance in this article.

In this work, we focus on natron glass dated to the eighth-second century BCE, and especially around the mid-1st millennium BCE in both eastern and western Eurasia. In a macroscopic consideration of Mediterranean natron glass, we suggest an encompassing framework based on trace elements for grouping natron glass of this time. Set in this technological context, we report the discovery of a natron-based glass bead from the Wupu cemetery, which is one of the earliest natron glass artifacts reported so far in East and Central Asia. Combining this analysis with the composition and typology of specific natron-based eye beads, we propose a production and circulation model for the dispersed natron glass products during this period. With these findings, we provide, to our knowledge, the first comprehensive discussion of natron glass dispersal across Eurasia in the 1st millennium BCE.

## The 1st millennium BCE natron glass in the Mediterranean

Natron and trona (simply referred to as natron below) are evaporite deposits consisting of mostly sodium carbonate. It is likely that most of the natron available was provided by Egypt, with the best-known source Wadi El Natrun about 100 km northwest of Cairo^17^. Archaeological evidence mainly from the Roman period shows that natron glass production involved a few primary glass-making centers, which produced raw glass from raw materials, and numerous secondary workshops, which re-melted raw glass ingots to shape into final products^18,19^. This situation should also apply to the 1st millennium BCE in general.

The history of natron glass began with a phase of trials and experiments in the early 1st millennium BCE, leading to the earliest variants of natron glass, such as the unstable low-Ca type, the high-Al cobalt-blue type and the high-iron black type^20–26^, which mostly had a low Ca content. In a few centuries, chemically stable natron glass containing a significant level of Ca (around 5–8%) emerged, and the main composition of natron glass stayed largely consistent afterwards. This ‘classic’ type of natron glass has been discovered from the 1st millennium BCE context across the Mediterranean area, including Italy^25–30^, Greece^31–34^, and Spain^35^, as well as in the Black Sea region with artifacts from Bulgaria^36,37^ and Georgia^38,39^ (a summary is given in the Supplementary Information (SI)). Moreover, natron glass was found outside the Mediterranean coastal area, such as Germany^40,41^, Poland^41,42^, and as far as Mali^43^. Intensified glass production in the late Hellenistic period^19,44^ eventually set the stage for Roman glass, when natron glass thrived as a focal center of material culture.

**Figure 1:**
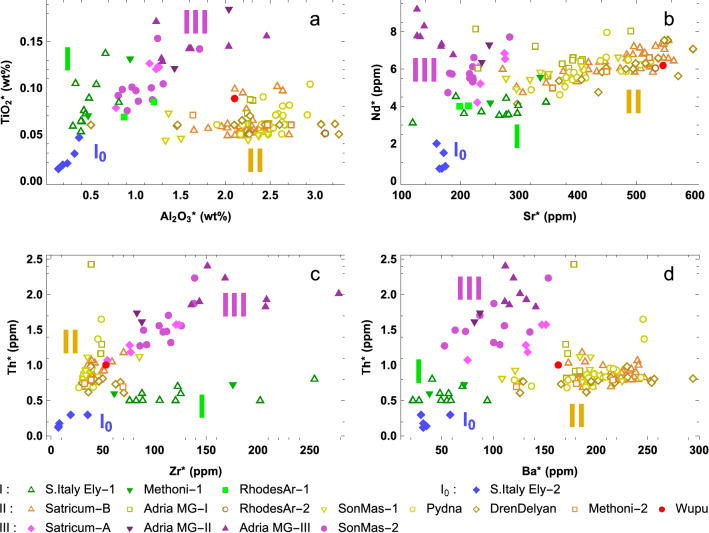
Chemical composition shows three major types (I, II, and III) and one minor type (I_0_) for natron glass dated to the 8–2 C. BCE. (**a**) TiO_2_ vs Al_2_O_3_. (**b**) Nd vs Sr. (**c**) Th vs Zr. (**d**) Th vs Ba. The samples are from: southern Italy’s early ‘classic’ natron glass (subgroup 1: 8–6 C.; subgroup 2: 8–7 C.)^25,26^; Rhodes, Greece, of the Archaic period (640–600, two subgroups)^31^; MG-II samples from Satricum, central-west Italy (4–3 C., clusters A and B)^30^; Adria, northern Italy (Mediterranean Group-I, -II, -III: 5 C., 3 C., 2 C., respectively)^29^; Pydna, Greece (6–4 C.)^32^; Methoni, Greece (6–4 C., two subgroups)^32^; Son Mas, Mallorca, Spain (subgroup 1: possibly 4–3 C.; subgroup 2: possibly 3 C.)^35^; Dren-Delyan, Bulgaria (end of 6—upper 4 C.)^36^; and Wupu, China (760–481, discussed in the next section). All dates are in BCE. All element data are normalized (marked with asterisks). Not every element is available for all samples.

To understand natron-based glass-making technology of this time, we examined the chemical composition of natron glass dated to after the initial exploratory phase and before the late Hellenistic period, which is approximately between the eighth and the second centuries BCE. This macroscopic analysis is based on the compositional data of 145 natron glass samples from 12 locations (see supplementary text and file). Based on principal component analysis (PCA), after examining 18 major, minor and trace elements we chose to investigate mainly using the trace elements Ba, Zr, Ti, Sr, Nd, Th, and La, which can be divided into three geochemical groups: Sr and Ba; Zr and Ti; Nd, La and Th. Only one major element, Al, shows the potential for effectively distinguishing different clusters of samples. The PCA treatment is described in the Methods appendix and in Supplementary Figs. S1 and S2. The PCA results and bivariate plots show that the 145 samples can be consistently categorized into three major compositional types denoted as I, II, and III, and one minor type denoted as I_0_ (Fig. 1, also Supplementary Figs. S2, S3, S4). Since some of the glasses are rich in lead and iron, all the elements (except Fe and Pb) are normalized to a composition from which iron and lead oxides have been removed (i.e. A^*^ = A/(1-PbO-Fe_2_O_3_) where A is an element's original concentration and A^*^ is the normalized concentration). When a site has diverse types of glass, we divided the samples into numbered subgroups. The four compositional types sometimes slightly overlap, probably due to glass recycling or mixed materials. Additional assemblages of natron glass dated to this period^27,28,33,34,37,38,42^ can also be attributed to these types, with details given in the SI. Supplementary Table S2 provides a statistical summary of element concentrations for each type.

The basic nature of the siliceous source for each type can be understood through a quick observation of the data (Table S2). The levels of Mg, K, and Al (Fig. 1a) provide an estimate for the silicate impurities, e.g. feldspar, mica, pyroxene, and amphibole. Types II and III are rich in Ca, and have Al and K levels much higher than the other types. Since quartz sand is usually rich in calcium carbonate and contains Al-bearing clay and feldspar, it was likely used to make the raw glasses for Types II and III. Type I_0_ is very low in Al, K, Fe as well as almost every trace element examined here, and contains higher Si than the rest, thus a pure silica source was used^26^, such as crushed and ground pebbles or vein quartz. Type I is high and variable in Ca, and low and slightly variable in Al, K, and Fe, indicating the use of clasts that contain lime and few impurities (e.g. feldspar) as the siliceous source. Types I and I_0_ are similar in composition, except that the latter contains fewer impurities. Within available data, most samples of Types I and I_0_ are relatively rich in Mn.

Trace elements Sr and Ba are related to lime, mica, and feldspar. Type II occupies the high-Sr, high-Ba range, in contrast to Types I_0_, I, and III (Fig. 1b,d). Sr substitutes for Ca atoms at different proportions in different minerals. A high Sr level (or preferably, Sr/CaO ratio) in the glass usually indicates the presence of aragonite (in the form of shells) in the glass-making sand from a coastal setting, while a low Sr content suggests the use of mainly calcite (as limestone debris) in the vitrifying material^45,46^. These could be the cases for Type II and for the other types, respectively. Ba can substitute for Sr atoms, and to a lesser extent for Ca atoms which are smaller in size. For Type II, Ba mostly derives from Sr-rich shells, which explains its overall higher Ba level. Limestone provides part of Ba for Types I and III. Also, potassium feldspar and mica introduce additional Ba into Type III (details in the SI).

The heavy mineral components reveal key information for provenance. Ti is often found in rutile, ilmenite, and titanite. Zr is strongly related to the presence of zircon, which usually first derives from weathered felsic igneous rocks such as granite in inland regions. Among the types, the Ti and Zr contents in Type II are the lowest (except Type I_0_) (Fig. 1a,c). Types I and III are higher in Zr, indicating siliceous sources from zircon-enriched deposits. The variable Zr and Ti ranges for Types I, I_0_, and III may be caused by a heterogeneous distribution of heavy mineral crystals, pointing to coarse-grain, less-sorted, continental rocks. Meanwhile, the compact ranges of Zr and Ti for Type II suggest that its raw glass had a stable supply of well-sorted beach sand with fine-grain, homogenized particles.

Certain rare earth minerals are indicative of the intrinsic trends in sediment. Nd is a light rare earth element (REE) often associated with monazite and allanite, which trace to clastic sediments and ultimately to the parent rocks in the source area. An almost linear Nd–Sr trend exists for Type II, and extends to some Type I samples (Fig. 1b). This correlation between the two divalent elements may result from the occurrence of allanite, which hosts both Nd and Sr. Th is often associated with heavy REEs due to their similar cation sizes in accessory minerals like zircon and monazite. Type III has exceptionally high Th values (Fig. 1c,d) compared to the other types, implying quite different species of heavy crystals such as different members of the monazite group for Type III. There is a positive Th-Zr correlation for Type III (Fig. 1c) either due to the presence of Th in the crystal lattice of zircon or the coexistence of zircon and monazite/allanite as accessory minerals. A distinct geological setting for the source area is therefore involved for the vitrifying material of Type III glass.

Based on the chemical composition, each type has a different origin for its siliceous source. Type II is similar, although not identical, to the Levantine-I type of Roman glass^47^. Roman-era written works have associated the Levant with glass-making activities: Strabo referred to Sidon in Lebanon as a Roman glass making center; Pliny the Elder proposed the mouth of Belus River as a source for glass-making sand^48^. Direct archaeological evidence for the primary production of pre-Roman natron glass is however very rare (one late Hellenistic site in Beirut dated to the 1st C. BCE has been discovered^19^). Considering all available information, the most likely location of the primary production for Type II is still the Syro-Palestinian coast. Indeed a Levantine origin for the raw glass has been suggested for samples of Type II^29,32,35–38,42^. Type III is rich in heavy minerals. Egypt is known for its heavy-mineral sands originally deriving from the upper Nile region^49^. The Nile River particles and the African dust are both high in Nd^50^, which agrees with Type III's distinct sediment signatures. Recently an Egyptian origin has indeed been proposed for the raw glass of certain Type III artifacts^34,35^. Interestingly, the high-iron black natron glass provides collateral support for this, as its siliceous source may be geographically related to Type III's (see the SI). For Type I, the low levels of impurities and the opposite trend of heavy minerals indicate that the quartz detritus used for glass-making did not originate from the same area as Type III. Except for a few later samples from Greece, most Type I artifacts were recovered from southern Italy or Central Europe. It is possible that these items were produced in Italy and were traded to the other side of the Alps by way of the Amber Road. Also, the unique Type I_0_ has only been found in southern Italy and is limited in sample quantity, which likely indicates a local raw glass production in the central Mediterranean (other possibilities are considered in the SI).

Curiously, the average soda content in Types I and I_0_ is higher than in the other types (Supplementary Table S2 and Fig. S3a), despite that they were made and circulated in areas far from the most prominent source of natron, i.e. Egypt. It is likely that with little Al, Fe, or K in the vitrifying material, more flux was needed to bring down the melting temperature of silica to a manageable level and to obtain a well-vitrified product. For Type I_0_ the lime content was likely added separately^26^. Although the siliceous source for Type I likely contains limestone fragments, additional limestone added separately could not be ruled out, considering some samples are quite rich in Ca but generally free of any other impurities. Type I's very high Ca and Na levels, which are higher than Type I_0_′s, thus probably reflect a continuity in technical procedures from Type I_0_. Moreover, a few coeval (7–6 C. BCE) black natron glasses uniquely utilized a siliceous source similar to Type I's, and their iron content was likely added separately, unlike earlier black glass^26^. These processes may indicate a proactive management of functional components, i.e. the ability to add and adjust individual components to match the siliceous source for a desired glass composition. For this to happen, it is pre-requisite to have a good understanding of the functions of different components and materials. This understanding did not exist for earlier black natron glass or high-Al cobalt-blue natron glass, in which Fe from the black sand or Al from the cobaltiferous alum automatically acts as the network stabilizer, unbeknownst to the glass-makers. Therefore, around the 8–7 C. BCE, glass-makers for the first time created the ‘classic’ natron glass by intentionally controlling the quantities of natron and lime added, a technological breakthrough in the history of natron glass. Although the use of Levantine coastal sand later eliminated the need for extra lime and slightly lowered the soda level, glass-makers were able to stick to the ‘classic’ recipe for high-quality natron glass with suitable levels of soda and lime ever since, regardless of changes in raw materials.

As a final note, we consider the chronological sequence of these types to identify overarching trends. I_0_ and I are the earliest types. Type I_0_ is mostly dated to the 8th C. BCE. Production of Type I glass may have started in the 8th C. BCE, and most artifacts are dated to before the 6th C. BCE. Since both Type I and Type II have been found from Rhodes (640–600 BCE)^31^, Methoni (6–4 C. BCE)^32^, and Poland (Hallstatt D)^42^, the transition between the two types probably took place in the 7–6 C. BCE. Type II has the largest number of reported samples among all types. Production of Type II glass possibly began in as early as the 7th C. BCE. The MG-I and some MG-II vessels were made using Type II raw glass. The dates of the samples from Greece and the Black Sea region^31–34,36–38^ suggest that from the middle to the late 1st millennium BCE and beyond Type II glass was supplied without interruption in the eastern Mediterranean. Type III artifacts were excavated from Italy and the western Mediterranean. Although they are dated to the 4–2 C. BCE, this date should be seen as region-specific. The production of Type III raw glass presumably started at an earlier time in Egypt, considering Egypt's accessibility to raw materials and the earlier black natron glass as a precedent. Type III includes MG-II and MG-III vessels. Because MG-II vessels from Italy can be made with either Type II or Type III glass, a change of raw glass supply for this region likely happened at the time of MG-II (4–3 C. BCE). As the central and western Mediterranean was connected to the eastern Mediterranean and North Africa through the Phoenician maritime trade, this switch from Type II to Type III likely reflects a shift of the trade route, since the Phoenicians were moving westward at the time. The export of Egyptian raw glass to the central-western Mediterranean was likely short-lived, since a return to Levantine raw glass later ensued for late Hellenistic and Roman glass.

By reconsidering chemical data of the Mediterranean natron glass dated to the 8–2 C. BCE, we have proposed a framework of compositional types with their diagnostic features. Although provenances for some of the samples have been individually suggested before, our new interpretation based on large datasets is organized in a data-centric and top-down approach to study important systemic trends. Using this framework as a guide, we will attempt to identify technological links for natron glass finds from China, which is far from where natron glass mainly circulated.

## Natron glass from Wupu and other sites in China

**Figure 2:**
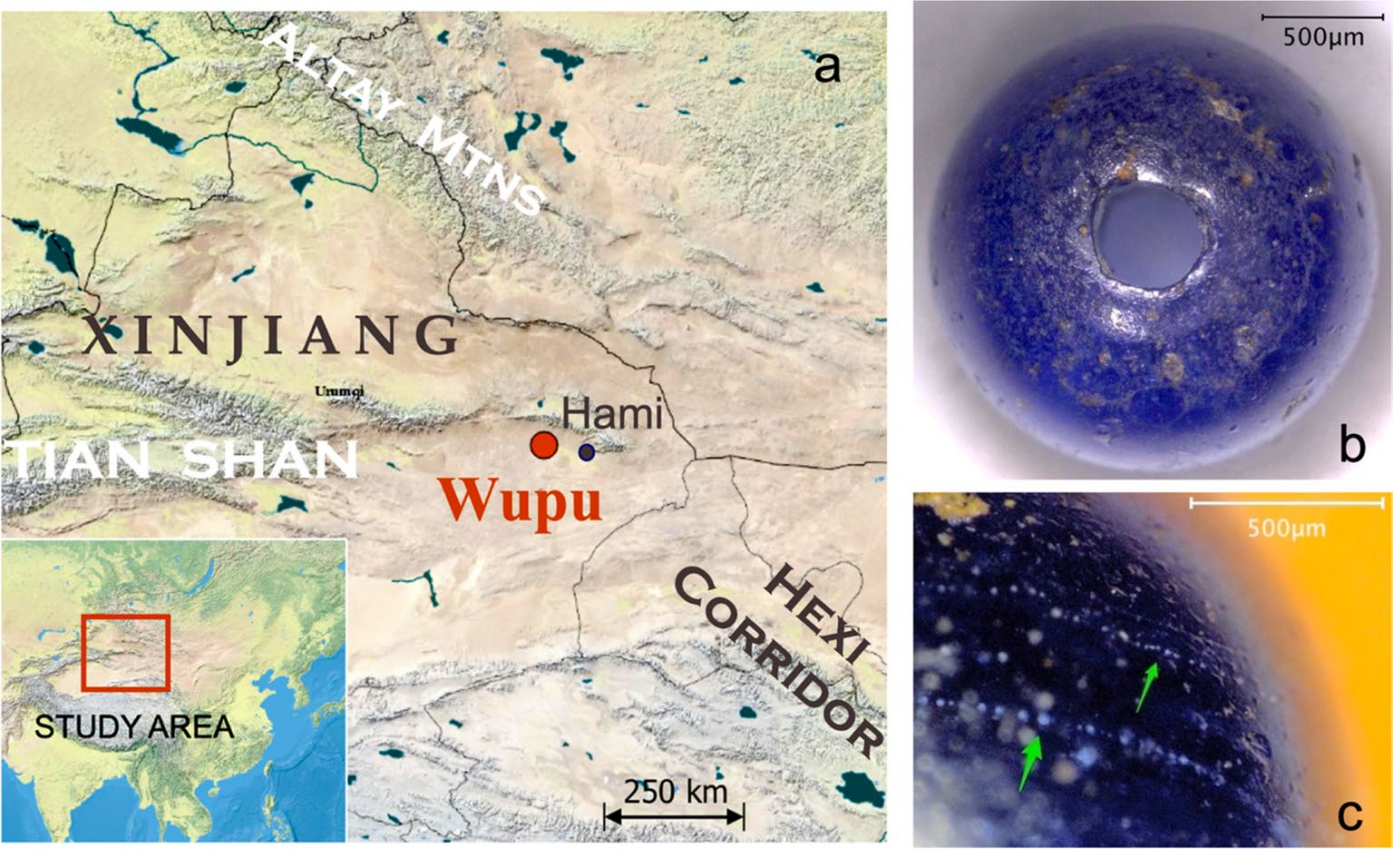
Information about the Wupu bead. (**a**) Wupu is located in eastern Xinjiang, near the eastern Tian Shan range. (**b**) The bead from Wupu. (**c**) Close-up view, chains of bubbles trapped in the glass are indicated. The map in (**a**) was created with QGIS 3.10 (https://qgis.org), based on the basemap of World Physical Map (http://goto.arcgisonline.com/maps/World_Physical_Map) with World Reference Overlay http://goto.arcgisonline.com/maps/Reference/World_Reference_Overlay).

The Wupu site is located at Kezierqueka, Wupu Township, Hami in eastern Xinjiang, 70 km from Hami’s urban area (Fig. 2a). It was settled from the Bronze Age to the Early Iron Age. Several glass beads have been discovered from the burials. Unfortunately, only one blue bead was available for us to analyze (Fig. 2b). Delimited by the latter phase of settlement, the bead is dated to 760–481 BCE. Wupu’s archaeological background is described in the SI.

This globular bead exhibits a deep blue color. Its surface is mostly smooth. Its body contains many bubbles, caused by gas released from decomposed ingredients during the manufacturing process. The bubbles are spherical rather than oblate or distorted, which indicates that the glass was likely not pulled or pressed. Also, no circumferential streaks from winding are on the surface. Thus the bead was not made by drawing, cutting or winding, and was most likely made in a mold. Some bubbles form chains (Fig. 2c), likely due to the following mechanism. After the bubbles were formed, they ascended in file to the surface. While larger bubbles reached the surface and escaped from the melt, some small bubbles were trapped inside the increasingly viscous melt as the temperature decreased. When the glass was shaped in the mold, the chains of bubbles gained the curvature of the bead shape. Confocal Raman spectroscopy displayed only two broad envelopes near 500 and 1000 cm^−1^ for the non-crystalline Si–O network, indicating the bead is well-vitrified.

The chemical composition of the bead was obtained by minimally destructive LA-ICP-MS analyses. The analytical method followed the protocol which involves no internal standards^51^, with details in the Methods appendix. For LA-ICP-MS, moderate surface corrosion is generally not a concern, since only data acquired after the initial ablation of surface layers are used. A consideration of the accuracy and precision of LA-ICP-MS analysis for archaeological glass can be found in Lü and Wu (2019)^52^. The calibrated data are listed in Table 1. Overall uncertainties are normally within 10%, except for Cr, Co, Ni, Zn, and Sn whose uncertainties are estimated at 15% due to a greater variation in their distribution in the glass. The Wupu bead is made of natron-based soda-lime glass. It has low levels of Mg and K, as well as a low phosphorus content and a presence of chlorine (1.35%, detected by semi-quantitative X-ray fluorescence), all of which are typical signs that natron was used as the alkali flux^17^. Cobalt in the Wupu bead is at a high level of 914 ppm. Co(II) is a powerful colorant and is responsible for the deep blue color of the bead. Antimony is at 0.6% in the bead, consistent with the Sb range in natron glass before 200 BCE^16^. Since the Wupu bead is semi-translucent, and no signature for the opacifying calcium antimonate was identified from Raman spectroscopy, we suggest that Sb is dissolved and functions as a fining agent.

**Table 1:** Chemical composition of the bead. Data are in wt % where marked with the percent sign, or in ppm when unmarked.

Na_2_O %	MgO %	Al_2_O_3_ %	SiO_2_ %	K_2_O %	CaO %
19.23	0.67	2.06	66.93	0.57	6.87

FeO %	P_2_O_5_ %	TiO_2_ %	MnO %	Co	Ba
1.87	0.037	0.087	0.0177	914.1	159.8

Cu	Sr	Sb	La	Cr	Pb
3553	534.4	6372	6.10	10.66	68.6

Zr	Zn	Ni	Sn	Nd	Th
52	135	44.91	9.25	6.06	0.98

Comparable samples from Wupu and China are severely limited, and we rely on a comparison with contemporaneous Mediterranean natron glass for provenance studies. The Wupu bead is classified as Type II (Fig. 1), suggesting it was manufactured with Levantine raw glass made from coastal sand. Pinpointing the exact location of secondary or final production with only compositional information is usually hard. To seek potential links, we note that most Type II artifacts were excavated from Greek, the Black Sea littoral, or Italian sites. During the 8–6 C. BCE the Greeks initiated a massive colonial expansion in the Mediterranean and the Black Sea regions^53^, and in the following centuries maritime trade within the Greek world was frequent. As this aligns with the geography and the chronology of Type II artifacts, it can be reasoned that Type II was probably closely related to Greek influence. At a time when Greek settlements dotted *Magna Graecia*, some of the Type II artifacts, including the Wupu bead, could have been produced in a Hellenized context in Italy. The large amount of Type II artifacts found in Italy and Central Europe dated to the mid-1st millennium BCE suggests that Italy was likely one of the supply centers of Type II products. The Wupu bead is also similar in trace elements to some of the Italian Type II samples. Certainly, there were glass workshops in other Greek settlements. One workshop in the Greek settlement of Yahorlyk (around the 6th C. BCE) in southern Ukraine was still making plant-ash glass, and likely only had a very limited supply of raw natron glass^54^ (but not of the Type II composition). This suggests that the Black Sea region was probably not a major producer of natron glass products. Therefore, it seems that the Wupu bead was more likely made in the central Mediterranean, but production in the east, e.g. a workshop in Greece, can't definitely be ruled out.

**Figure 3:**
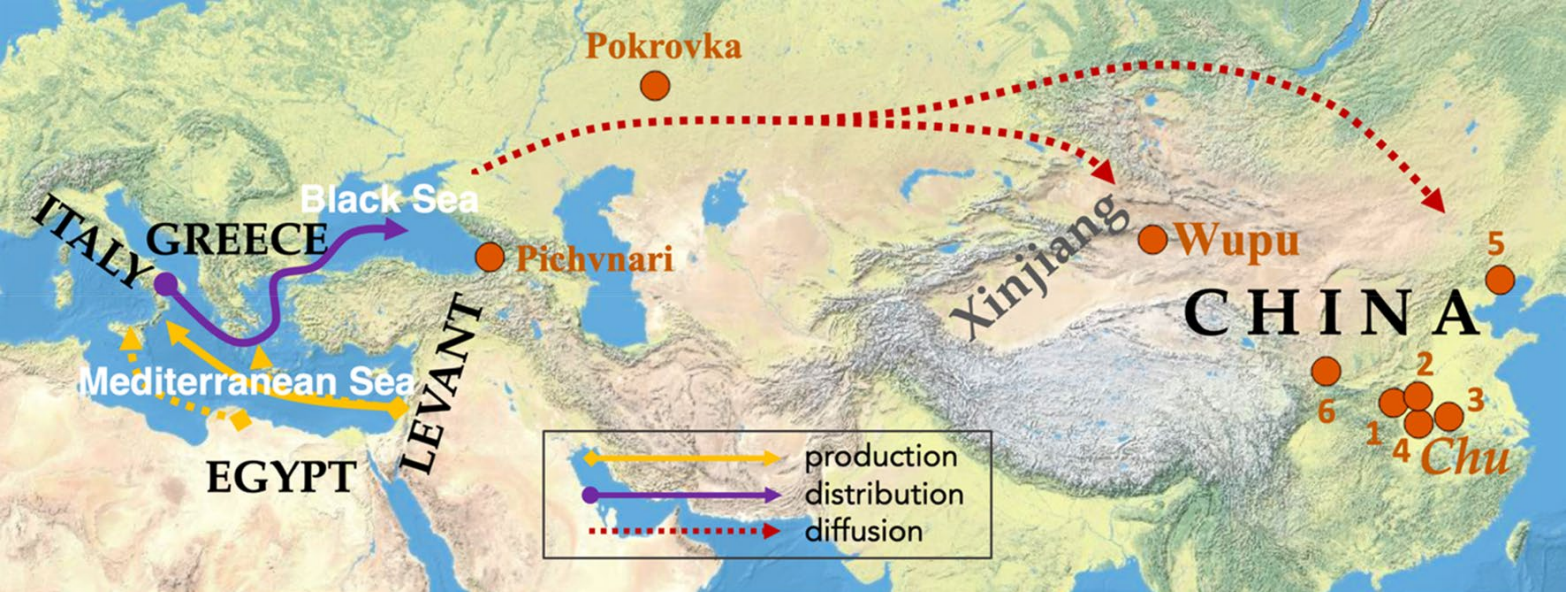
The three-segment ‘life history’ model for the dispersed natron glasses. Production (yellow line): raw glass was shipped from the Levant to workshops across the Mediterranean for secondary production, with one likely route for Type II glass indicated by the solid line. Egypt also provided raw glass but not likely for the products traveling to eastern Eurasia. Distribution (purple line): finished glass products were traded in the Mediterranean and the Black Sea regions as Greek colonization intensified. Diffusion (red line): small glass jewelry was carried by nomadic groups while traveling across the Eurasian Steppe, who essentially facilitated the eastward flow of natron glass. Indicated routes are not meant to be exact. Sites to the east of the Black Sea where natron glass dated to the mid-1st millennium BCE have been found are marked by orange dots. Chinese sites with early natron glass: central (Chu state): 1 Xujialing, 2 Jiuxian, 3 Hougudui, and 4 Zenghouyi/Leigudun; north: 5 Jianchang; west: Wupu and 6 Majiayuan. This map was created with QGIS 3.10 (https://qgis.org), based on the basemap of World Physical Map (http://goto.arcgisonline.com/maps/World_Physical_Map).

Because there is neither any natron exploitation for glass-making nor discovery of raw natron glass in East and Central Asia, all early natron glasses in China were imported as final products. The Wupu bead is one of the earliest natron glass artifacts reported from East and Central Asia, and possibly the earliest. Its analysis also provides the first high-quality data for natron glass in this region. Before this study, the eye beads from Jiuxian^55^ and Xujialing^56^ in Henan Province, and Zenghouyi^57^, Suizhou, Hubei Province, dated to the late Spring and Autumn (S. & A. 771–476 BCE) and the early Warring States (W. S. 475–221 BCE) periods, have been suggested as natron glass. Particularly, in Zenghouyi as many as 173 beads were discovered. Natron-based eye beads dated to the W. S. were also recently announced from Dongdazhangzi in Jianchang, Liaoning Province^58^. Moreover, some of the artifacts previously reported simply as soda-lime glass are likely natron-based. According to published data, we suggest that natron glass has actually been recovered in Hougudui (late S. & A.) in Gushi, Henan, central China^59^, Leigudun Tomb II (W. S.) in Suizhou, Hubei, central China^60,61^, and Majiayuan (late W. S.) in Tianshui, Gansu, central-west China^62^. All these artifacts are eye beads. It should be noted that high-quality data of pristine un-corroded glass are desired for detailed provenance research. Data availability and quality vary for these analyses. For many elements, quantitative information was either affected by severe weathering or absent due to restrictions from analytical methods. We empirically identified potential natron glass from available data, and concluded that these beads exhibit major element levels characteristic of the ‘classic’ natron glass (see the SI). The Chinese sites where potential natron glass was found are marked in Fig. 3. Other than the finds from Dongdazhangzi and Majiayuan, the rest were excavated from areas that belonged to or were heavily influenced by the Chu state.

Due to the constraints on available data, we adopted a hybrid compositional-typological approach to further investigate natron-based stratified eye beads from China. Eye beads are so named because smaller spots of one color are superimposed on top of larger spots of another color, resembling eyes. On the surface of the bead's *body*, these are shown as *inlaid areas* that contain concentric *rings* surrounding the *pupil*. Previous review of eye beads in China studied the bead forms and the eye shapes^63^, but has not focused on colors. Colors of different components indicate glass materials available to the bead craftsmen, and by extension the coloration techniques of related workshops. Here, among the globular stratified eye beads across Eurasia and dated to around the mid-1st millennium BCE, we identified four typologies mainly by the colors of the components, denoted as EB-a to EB-d, based on published images. Each eye bead type has a distinct combination of colors for the body, pupil, ring and inlaid areas (Table 2), and they also differ in the arrangement of the eyes.

**Table 2:** Four types of mid-1st millennium BCE stratified eye beads across Eurasia. Parentheses indicate probable cases. Likely composition for a type is inferred from samples of that type with trace element information reported. Sites of discovery: EB-a: Dren-Delyan (Bulgaria)^36^, Pichvnari (Georgia)^39^, the tomb of Zhaoqing (north China)^64^, Zenghouyi (central China)^57^, and Shanghai Glass Museum (unspecified context)^64^; EB-b: Altdorf (Germany)^41^, Owidz (Poland)^42^, Dren-Delyan^36^, Pichvnari^39^, National Museum of Georgia (unspecified context, personal visit), Zenghouyi^57^, and Shanghai Glass Museum^64^; EB-c: Xujialing (central China)^56^, Zenghouyi^57^, Hougudui (central China)^15^, and Pichvnari^39^; EB-d: from Son Mas (Spain) as fragments^35^, and from Svaneti Museum in Georgia (unspecified context, personal visit), National Museum of Georgia, and Shanghai Glass Museum^64^. Additionally, eye beads that might be of EB-a, EB-c, and EB-d types were reported from Filippovka I burials in Southern Urals that were considered to date to 4–3 C. BCE (images inconclusive^65,66^).

Typology	Body	Pupil	Ring	Inlaid area	Regions of discovery^15,35,36,39,41,42,56,57,64,65^	Likely composition
EB-a	greenish-blue	deep blue	deep blue	white	N. China, C. China, (Russia), Georgia, Bulgaria	Type II natron
EB-b	yellow	deep blue	deep blue	white	C. China, Georgia, Bulgaria, Poland, Germany	Type II natron
EB-c	green/blue	deep blue	brown	white	C. China, (Russia), Georgia	natron
EB-d	deep blue	deep blue	none	white	China, (Russia), Georgia, Spain	Type II natron

Some of the beads have compositional data reported. The Altdorf beads^41^ (EB-b) are made of natron glass, and the beads from Zenghouyi^57^ (EB-a, -b, -c), Xujialing^56^ (EB-c), Hougudui^15^ (EB-c) and Shanghai Glass Museum^64^ (EB-a, -b, -d) are likely natron-based as well. Also, Pichvnari beads that may be of EB-b and EB-d types from textual descriptions were reported as natron glass^38^. Notably, trace element data are available for beads from Dren-Delyan^36^ (EB-a, -b), Owidz^42^ (EB-b), and Son Mas^35^ (EB-d), all of which belong to Type II. Beads of the same typology likely also possess identical or very similar composition. We thus believe that all four eye bead types are made of natron glass, and that at least EB-a, EB-b, and EB-d are of Type II composition, the same type as the Wupu bead. Therefore, it is reasonable to suggest that these eye beads are compositionally comparable to Greek-Italian natron glass.

Coloration of the base glasses attests to a shared technological background. Among these eye beads, glass of the same color contains the same colorants and opacifiers: white glass is rich in Sb and Ca, indicating the presence of calcium antimonate^35,36,41,42,56,57,64^; blue or greenish-blue glass is colored by Cu^36,56,57,64^; Sb and Pb are high in yellow glass that is colored by lead antimonate^36,41,42,57^; deep blue glass is colored by Co and mostly also contains some Cu^35,36,41,42,56,57,64^, similar to the Wupu bead.

Similarity in typology and composition strongly suggests a common origin. From these common characteristics, we propose that around the mid-1st millennium BCE, most of the eye beads were made by a number of specialized craftsmen with a limited variety of somewhat standardized base glasses, which were supplied by major workshops around the Mediterranean through a developed network. Since all four types have been discovered along the Black Sea coast and in China in the 5–4 C. BCE, we suggest that the glass items China received were strongly dependent on what the Black Sea region was able to obtain, and that a large amount of Mediterranean natron glass beads may have entered central China by the early Warring States period.

## Natron glass and the Proto-Silk Road

For natron glass products to reach Xinjiang and central China, an extensive network of trade or exchange must have existed. The Eurasian Steppe is an expansive and continuous ecological zone spanning from Eastern Europe to Siberia, and had been proposed as a corridor linking major civilizations of Eurasia^10,67,68^. The Steppe could have provided the route for the dispersion of Mediterranean glass. The connection routes between the western and eastern Eurasia that preceded the historical Silk Road can be suggested as the ‘Proto-Silk Road’. Figure 3 displays a schematic model for the ‘life history’ of the dispersed natron glass items against the backdrop of the Proto-Silk Road. The sites along the Steppe where natron glass has been found are marked in Fig. 3. These finds, although still sparse, suggest possible ways the Mediterranean was connected with China through the Steppe.

The first segment of the Proto-Silk Road was pertinent to the links between the production sites and likely involved highly active sea-faring groups such as the Phoenicians. Raw glasses from Syro-Palestine, Egypt, and possibly other areas were brought to secondary workshops across the Mediterranean, and specifically Italy in this research’s context, as illustrated by the ‘production’ lines in Fig. 3. To manufacture natron glass, not only were resources from different places drawn together (commodity trade), also specialized expertise to treat glass at different stages was developed locally (division of labor). This inter-regional collaboration may be deemed as a prototype of the ‘supply chain’, testifying to an early glass industry.

The finished glass products catered to an integrated Greek market as well as other Mediterranean populations. The trade between workshops and consumers was akin to multi-channel sales of merchandise, signified as the ‘distribution’ segment in Fig. 3. Greek expansion also promoted the spread of Mediterranean material culture to the Black Sea, bringing Mediterranean goods in contact with native groups^53^. Blue-and-white eye beads are frequent finds in Scythian burials near Greek colonies along the Black Sea^69^. Museums in Georgia house a large number of blue beads and blue-and-white eye beads from Colchian and Greek settlements (personal visit).

Eastward from the Black Sea, nomadic groups roamed the Steppe, who likely carried small decorative items with them. Natron glass beads from burials dated to the early Sarmatian period (4–2 C. BCE) were found in Pokrovka, Orenburg, Russia, which are similar in style to beads from Scythian sites in Crimea and the Don Basin^69,70^. The Pokrovka beads include monochrome blue beads, blue-and-white eye beads, and a black glass bead, representing several types popular in the Mediterranean. The eye beads and core-formed glass vessels excavated from the Filippovka I burials^66^ also testify to the position of Southern Urals in the movement of Greek and Mediterranean glass items. Furthermore, from the 1st millennium BCE context, high-alumina soda glass has been found in Pokrovka and Pichvnari, and potash glass has been found in northwestern Xinjiang and Pokrovka^38,69,71^, which likely originated from South Asia. These finds demonstrate the Steppe's role in connecting diverse sources and facilitating specific diffusions oriented to either directions. The long-distance dispersion of glass jewelry was unlikely to have occurred in a single step. It was argued that the pastoralists might have moved goods with their seasonal migration, essentially assuming the role of intermediaries^69^. Nomadic migration routes have been proposed as the predecessor of Silk Road in the mountainous areas of Inner Asia^72^, and there could be similar dynamics for long-range connectivity across the Steppe. It was also possible that the internal population interflows of eastern and western Scythian groups during the 1st millennium BCE^73^ had promoted the spread of material culture. Importantly, although not involving agents such as diplomats and specialized traders later traveling along the Silk Road, the movement of early natron glass along the Proto-Silk Road was highly directional and relatively fast. This indicates that around the mid-1st millennium BCE, some form of integration of the intergroup communication network probably had taken place. Glass jewelry was part of luxury decorative objects which were often displayed as symbols of social or political status, and the demand for these objects could have driven intergroup exchanges. It has been suggested that an interconnected network involving a small number of highly visible elites could be sufficient for the propagation of exotic valuables across great distances^10^. Although the intergroup interactions might initially start with the trade or exchange of small quantities of prestige goods, such processes progressively reinforced the connections and improved the efficiency of inter-regional communications, eventually leading to trade activities of economic significance and the expansion of interaction spheres. The Steppe routes are represented by the ‘diffusion’ lines in Fig. 3.

Since the Bronze Age, eastern Xinjiang, where Wupu is located, was an intermediate junction connected to both the Hexi Corridor in the east and the Steppe in the north. The site of Tianshanbeilu in the Hami region, dated to the 2nd millennium BCE, has pottery and bronze similar to those from the Siba culture in the Hexi Corridor^74,75^. Eastern Xinjiang also has routes open to the Steppe, and northwestern Xinjiang may have contributed to this link. Soda-enriched imported faience beads have been excavated from the Ya'er cemetery^76^ near Wupu. Some bronzes discovered in the Hami region were possibly made with ores from Ili in northwestern Xinjiang^77^. Mixed-alkali faience beads have been reported from Tianshanbeilu^78^, while similar mixed-alkali faience beads dated to the 19–15 C. BCE and suggested to originate from Europe have been discovered in an Andronovo-influenced site in Wenquan, western Xinjiang^79^. Additionally, it should be noted that Xinjiang is also accessible via the mountain valleys from Central Asia. Increased precipitation had created favorable conditions for social development in Central Asia in the 2nd millennium BCE^80^. Agricultural intensification and sociopolitical changes in Central Asia^81^ and the development of the city-states of the Tarim Basin^82^ have also been suggested for the 1st millennium BCE. However, material culture evidence signifies early interactions of eastern Xinjiang with the Steppe. A dearth of natron glass artifacts reported from Central Asia, as well as the later dates of natron glass from the oasis sites than eastern Xinjiang and central China^83–86^ currently does not associate the diffusion of early natron glass with this alternative route, although this understanding may be updated by future archaeological discoveries.

North-south movement between the Steppe and north-central China is also viable, and material culture provides much evidence of this interaction, such as bronze weaponry and horse-drawn chariots^87^. This is corroborated by the early 1st millennium BCE faience beads with soda-enriched glaze excavated from Shanxi Province in north China, which may originate from the Near East^88^. Although it is unclear how the Chu elites in central China were connected to their northern Steppe counterparts, and a direct connection seems unlikely, Chu, being one of the major powers in the mid-1st millennium China, was certainly very capable of acquiring exotic valuables, thereby indirectly driving their movement. The fact that the natron glass beads found in central China are eye beads may reflect a cultural or aesthetic preference for eye beads by the Chu people. Early natron glass could enter central China in two possible ways: either through Xinjiang and by way of the Hexi Corridor, or from the Steppe directly. For early natron glass, no monochrome beads have been reported from central China, and no eye beads have been reported in Xinjiang so far. This seems to favor the possibility that both areas were in direct contact with the Steppe. It is also possible to conjecture that more than one chain of diffusion existed and the eastward movement of material culture diverged at the Altay Mountains.

Central China procured decorative glass items from a variety of sources. Glasses made with different technologies were sometimes found in the same burial, such as natron glass and plant ash glass in Zenghouyi^57^, and natron glass and potash glass in Leigudun Tomb II^60^. The great diversity of early glass found in China may reflect its initial status as a receiver rather than a producer of glass. The need for translucent decorations probably encouraged the first Chinese glass-making by imitating foreign glass, especially eye beads. Eye beads made of lead-barium glass^89,90^ and potash glass^91^ soon began to appear in the Warring States burials. Before achieving successful glass-making, trials must have taken place. If imported glass had an impact on the invention of Chinese glass-making, natron glass was probably used as a technological reference since early Chinese glass-making also utilized mineral-sourced fluxes. Previously published data included glass containing both lead-barium and a significant level of sodium and sometimes also lime^15,92^, signaling a possible intermediate step in the development of glass-making or a recycling practice. However, unlike natron glass in the West, no variants reflecting experimental compositions lasting over centuries existed for Chinese glass. Without prior experiences in making glass, Chinese craftsmen were soon able to ascertain proper conditions to manufacture glass using local materials. This seemingly abrupt inception of Chinese glass-making implies that a comprehension of functional components must have been in place. This perhaps partly owed to the example of proto-porcelain glazing as previously suggested^93^. It is also possible that as glass gained broader recognition in the Mediterranean, a basic understanding of the technological nature of glass traveled by word of mouth along with the steady flow of Mediterranean glass products and quickly resonated with existing Chinese ceramics and possibly bronze-making skills.

In the following centuries, as the Mediterranean glass-making technology evolved, the numbers of large monochrome beads and eye beads started to dwindle, and as a result they also became scarce in central China during the Han Dynasty, showing transcontinentally connected glass markets. Meanwhile, the trade routes going through the oasis states in Xinjiang and the Hexi Corridor became more prominent in the Han Dynasty. Through what is known today as the Silk Road, more natron glass flowed into China, many of which were Roman and Sasanian glass vessels^94^. Lead-barium glass also appeared in Xinjiang^86,95^, reflecting increased Chinese influence.

## Conclusion

In this work, we have assessed the 1st millennium BCE natron glass and examined the early eastward dispersion of natron glass across the Eurasian Continent. Among the four technological types of Mediterranean natron glass identified here, Type II is arguably the most influential type of natron glass in the East, as seen in the analysis of one natron glass bead from Xinjiang, and most if not all of the four typologies of natron eye beads that arrived in China from the Mediterranean and the Black Sea. For natron glass dispersal, we have proposed a three-segment model, which involves an ancient ‘supply chain’, a distribution network, and a swift intergroup relay at each stage. As the glass beads traveled eastward, they transformed from common merchandise to exotic symbols of status, thereby forming part of a prestige goods economy which in turn facilitated the integration of intergroup networks.

The picture we furnish the readers with is far from complete. Incorporating isotope analysis and taking colorants and opacifiers into consideration are next steps in our future research. Our discussion is inevitably limited by available samples and dates. The study of production locations will be improved by discoveries of archaeological evidence for production activities. As there is a large geographical gap between East Asia and the Black Sea, future analysis of glass excavated from Russia, Kazakhstan, and Mongolia will be immensely beneficial for piecing together the macroscopic picture.

The Proto-Silk Road, like the historical Silk Road, is a network of pathways to accommodate the East-West communication. As this work suggests, before the rise of the Chinese and Roman empires, a sustained connection along the Steppe may have resulted in the arrival of Mediterranean natron glass of mid-1st millennium BCE composition in Xinjiang before the early 5th C. BCE, not much later than its appearance in the Mediterranean. Increased quantities of natron glass beads reached central China by the first half of the 5th C. BCE, which possibly instigated the beginning of native glass-making in China. The flow of natron glass beads represents a relatively quick and unobstructed propagation of material culture along the Proto-Silk Road, which was by no means the result of random movement or sporadic exchanges. Our research illustrates an increasingly efficient and interactive transcontinental link in the 1st millennium BCE, which made possible an upcoming era of intensified communications and specialized trade.

## Methods

### PCA and the choice of elements for analysis

To determine if the samples can be successfully clustered, we processed the compositional data with principal component analysis (PCA) and considered the role of different elements. We examined the data of the following 18 elements: Si, Na, Mg, Al, K, Ca, Fe, Pb, Ti, Ba, Zr, Sr, Nd, Mn, Cr, V, Th, La. The obtained 1st and 2nd principal components (PCs) account for a total of 45.2% variance (23.6% and 21.6% respectively). The individual data points can be successfully grouped into four clusters: I, II, III, and I_0_. The elements with the largest contribution to the PCs are Sr, Ba, Th, Al, Nd, Ti, closely followed by Zr and La. The elements correlating the most with PC1 are Th and Nd, and the elements correlating the most to PC2 are Sr and Ba. The result is in general agreement with a mineralogical understanding of the roles of different elements in glass composition: trace elements are the most hopeful for indicating source minerals in raw materials; With most major elements, the contrasting trends are not easily discerned, since the common fluctuations of mineral contents may cause variations of major element concentrations; Most REEs have been suggested to display largely uniform patterns which cannot indicate provenance^18,47^, because the origins of these REEs are obfuscated with multiple contributing minerals; However, certain light REEs are notable exceptions and can signify the presence of accessory minerals. From PCA results, we chose to use the trace elements Ba, Zr, Ti, Sr, Nd, Th, and La and one major element Al for the analysis. Supplementary Fig. S1 exhibits the correlation of the elements with PCs, indicating that these eight elements are the best-represented in the PCs and thus qualify for distinguishing different sample clusters. All of these elements are associated with the siliceous source, thus their bivariate plots provide vital information about primary production. From the mineralogical point of view, the trace elements can be divided into three geochemical groups: Ba and Sr, usually associated with lime, mica or feldspar; Zr and Ti, both related to heavy minerals; Nd and La, which are light REEs and also linked to certain heavy mineral contents. Th often co-exists with REEs and heavy minerals, and shares traits of the latter two groups. Major element Al is related to the silicate impurities such as feldspar. After reducing the dataset to the chosen elements, we treated the data again with PCA to ensure the clustering of samples is consistent. The first two PCs now explain 83% of the total variance. Supplementary Fig. 2 shows the four groups identified by PCA. To display the grouping results with mineralogical and archaeological relevance, we decided to plot the following bivariant relations, which consistently shows the four compositional types: Fig. 1 (a) Ti vs Al; (b) Nd vs Sr; (c) Th vs Zr; (d) Th vs Ba; Supplementary Fig. S3 (a) Al vs Na; (b) Sr vs Ca; (c) Sr vs Ba; (d) Ti vs Ba; (e) K vs Ba; (f) Zr vs Ba; Supplementary Fig. S4 (a) 1/Zr vs 1/Ti; (b) Ti vs Nd; (c) Sr vs Ti; (d) Zr vs Nd; (e) Th vs Nd; (f) Th vs Sr; (g) La vs Zr; (h) Th vs La. To better illustrate different clusters, we typically use elements from different geochemical groups for a bivariate relation, although some combinations of elements were chosen for the purpose of invoking discussion in the text. On a side note, we found that if most trace element data are unavailable, it is often possible to speculate the compositional types with Al and Ti levels, although the result without corroboration from trace elements should be taken with a grain of salt.

### LA-ICP-MS

LA-ICP-MS analysis was conducted at the CAS Key Laboratory of Crust-Mantle Materials and Environments, University of Science and Technology of China^96^. The sample was first cleaned with an ultrasonic cleaner to remove any dirt on the surface. An Agilent 7700e ICP-MS instrument was used to record signal intensities in combination with a GeolasPro ArF (193 nm) excimer laser sampling system. The ablation spot was 44 μm in aperture size and the repetition rate was 10 Hz. Helium was used as the carrier gas and was flowing at 900 ml/min, and argon was the make-up gas which was mixed with the helium gas before entering the ICP. For each analysis, following a background acquisition of approximately 20 s, laser ablated the sample for a duration of 40 s for data acquisition, then followed by approximately 35 s for gas flow washing. We adopted the procedures described by Liu et al.^51^ for calibration with multiple external standards and no internal standard. This calibration protocol calculates the ablation yield correlation factor (AYCF) by normalizing the metal oxides total to 100%, which eliminates the need for using internal standards. The sequence began and ended with analyzing five reference materials (NIST SRM 610, NIST SRM 612, BHVO-2G, BCR-2G, and BIR-1G). NIST SRM 610 was analyzed periodically for time-drift correction. Data were processed with the ICPMSDataCal software^51^. A total of five spots on the surface were analyzed, and calibrated data were averaged to obtain the final values. The uncertainty of each element concentration is a combination of systematic error for each datum from one ablation, and standard deviation from multiple shots.

## Supplementary Information

Supplementary Information.

Supplementary Data.

## Data Availability

All the data used to support the analysis of this research are reported in this manuscript or have been published in the cited sources. A compilation of these data is also included as a supplementary spreadsheet file.
