# Supplementary material for: Natron glass beads reveal proto-Silk Road between the Mediterranean and China in the 1st millennium BCE
Source: Sci Rep. 2021 Feb 11;11:3537. doi: 10.1038/s41598-021-82245-w (PMC7878486; doi:10.1038/s41598-021-82245-w)
Supplement: Supplementary file 1 — Supplementary Information. [file 41598_2021_82245_MOESM1_ESM.docx]

Supplementary information for

**Natron glass beads reveal proto-Silk Road between the Mediterranean and China in the 1st millennium BCE**

**Qin-Qin Lü**^*^**, Julian Henderson, Yongqiang Wang, and Binghua Wang**

* E-mail: QinqinL@ustc.edu.cn or QLUPhys@gmail.com

**Materials**

**Sample data.** The samples included in our analysis are listed in Table S1, along with the sources. These samples range from the emerging ‘classic’ natron glass in the early 1st millennium BCE to the flourishing Hellenistic glass in the late 1st millennium BCE. Not all elements are available for every sample. Those for which compositional data of key elements are unavailable are not used. Samples from the same location or area may consist of more than one compositional type, in which case they are divided into sub-groups. For the sub-groups, we use the notations from the publications, if available, such as -A, -B, or Mediterranean Group (MG)-I, -II, -III. If such grouping is not available or does not fit our criteria, we divide the original assemblage into numbered sub-groups (i.e. -1, -2). When reviewing the clustering results from PCA and bivariant plots, we also took information from the given typology and assemblage into consideration, so that the division of data also aligns with archaeological categorization, and we found a good agreement. For a tiny fraction of samples that lie on the borders of different Types, we attributed them to the most possible group by examining the PCA result, different bivariant relations, typology assessment, and archaeological connotations. Some element concentrations (Th, Cr, V, Mn) are marked as below detection limit (bdl) or not available (NA) for a number of samples in the original publications. Since the detection limit is often not reported, we treated bdl as zero. For the cases of NA, we replaced the entries with the median concentration for the compositional type the sample belongs to (we also tested replacing them with zero to ensure the grouping results or the best-PC-correlated elements do not change). A compilation of the data is included as a supplementary file. A statistical summary, including the median, average, and standard deviation of the element concentrations for each compositional type is given in Table S2.

**Principal Component Analysis (PCA).** The PCA treatment is described in the Methods appendix of the manuscript. The process was conducted using R and the results were visualized with the *factoextra* package. Here, Fig. S1 shows the 18 elements under consideration in a correlation circle. Elements with better representation in the PCs are displayed as longer arrows that are closer to the circumference. It is clear that the elements Ba, Zr, Ti, Sr, Nd, Th, Al and La are the best represented. Fig. S2 shows the four groups obtained by PCA for the reduced dataset of the eight elements, which is consistent with the clustering result for the original dataset and in the bivariate plots. The four groups correspond to the four compositional types described in the main text: I, II, III and I_0_.

**Table S1. Samples included in the analysis.**

| Site | Subgroup | Date (BCE) | Quantity | Type | Source/Note |
| --- | --- | --- | --- | --- | --- |
| Wupu, China |  | 760-510 | 1 | II | this study |
| Satricum, Italy | Satricum-A | 4-3 C. | 5 | III | ^1^ |
| Satricum, Italy | Satricum-B | 4-3 C. | 39 | II | ^1^ |
| Rhodes, Greece | RhodesAr-1 | 640-600 | 2 | I | ^2^ |
| Rhodes, Greece | RhodesAr-2 | 640-600 | 1 | II | ^2^ |
| Methoni, Greece | Methoni-1 | 6-4 C. | 2 | I | ^3^ , note1 |
| Methoni, Greece | Methoni-2 | 6-4 C. | 3 | II | ^3^ , note1 |
| Pydna, Greece |  | 6-4 C. | 22 | II | ^3^ , note2 |
| Adria, Italy | Adria MG-I | 5 C. | 11 | II | ^4^ |
| Adria, Italy | Adria MG-II | 3 C. | 2 | III | ^4^ |
| Adria, Italy | Adria MG-III | 2 C. | 7 | III | ^4^ |
| Dren-Delyan, Bulgaria |  | end of 6 C. - upper 4 C. | 15 | II | ^5^ , note3 |
| Francavilla Marittima, Italy | S.Italy Early-2 | 8-7 C. | 3 | I_0_ | ^6^ |
| Francavilla Marittima, Italy | S.Italy Early-1 | 8 C. | 1 | I | ^6^ |
| Sarno(S. Valentino Torio), Italy | S.Italy Early-2 | 725-700 | 1 | I_0_ | ^7^ |
| Sarno(S. Valentino Torio), Italy | S.Italy Early-1 | 725-550 | 8 | I | ^7^ , note4 |
| Sarno(S. Marzano), Italy | S.Italy Early-1 | 750-725 | 1 | I | ^7^ |
| Capua, Italy | S.Italy Early-2 | 770-745 | 1 | I_0_ | ^7^ |
| Amendolara, Italy | S.Italy Early-1 | 7-6 C. | 1 | I | ^6^ |
| Son Mas, Spain | Son Mas-1 | maybe 4-3 C. | 8 | II | ^8^ , note5 |
| Son Mas, Spain | Son Mas-2 | maybe 3 C. | 11 | III | ^8^ , note5 |
| Total |  | 8-2 C. | 145 |  |  |

Only samples with complete or almost complete trace element data are included.

Note 1: Among the Methoni samples reported, those with trace element data are dated to 6-4 C. BCE. There are other samples with earlier dates.

Note 2: Two samples from Pydna (6267a, 8372) are not included due to potential discrepancies with data published in the ARCHGLASS database ^9^. One sample from Pydna (871) is not included since it contains very high Pb (36%) and is possibly affected by excessive additions. The sample is also listed as a special case in the source publication.

Note 3: Four samples from Dren-Delyan are not included: B beige (possible corrosion), 159 (black glass), 1182, and 1247 brown (extremely low Al, K, and Ba levels, some of which lie out of 3 times standard deviation from the Type II average).

Note 4: Two samples from S. Valentino Torio, Italy (SN3inc, SN38t) are not included since they contain extremely high levels of Ti, Mn, Zr, Cr, and some REEs, some of which lie out of 3 times standard deviation from the average of Type I or I_0_ (or any of the types identified here).

Note 5: Samples dated to after the 2nd C. BCE are not included. The attribution of samples differs slightly (one sample 3b2 is different).

**Table S2. Statistics for the composition of Mediterranean natron glass types.** The original compositional data used for statistical calculation are from sources listed in Table S1. Element concentrations with an asterisk (*) are normalized. Data are in wt % for oxides, or in ppm for elements. Not every element is available for all samples. NA = not applicable (due to sample size).

|  | median Type I_0_ | median Type I | median Type II | median Type III | average Type I_0_ | average Type I | average Type II | average Type III | std dev Type I_0_ | std dev Type I | std dev Type II | std dev Type III |
| --- | --- | --- | --- | --- | --- | --- | --- | --- | --- | --- | --- | --- |
| SiO_2_^*^ | 74.63 | 69.66 | 69.91 | 71.61 | 74.32 | 70.08 | 69.74 | 70.95 | 1.05 | 1.74 | 2.75 | 2.53 |
| Na_2_O^*^ | 17.83 | 18.80 | 17.43 | 15.48 | 18.34 | 19.04 | 17.43 | 15.71 | 0.84 | 1.24 | 1.32 | 2.19 |
| MgO^*^ | 0.44 | 0.54 | 0.54 | 0.49 | 0.55 | 0.62 | 0.54 | 0.50 | 0.22 | 0.19 | 0.14 | 0.17 |
| Al_2_O_3_^*^ | 0.24 | 0.47 | 2.30 | 1.25 | 0.25 | 0.58 | 2.31 | 1.32 | 0.094 | 0.26 | 0.40 | 0.41 |
| K_2_O^*^ | 0.23 | 0.15 | 0.53 | 0.58 | 0.26 | 0.16 | 0.57 | 0.63 | 0.076 | 0.066 | 0.22 | 0.32 |
| CaO^*^ | 6.03 | 8.20 | 7.25 | 7.19 | 5.98 | 7.65 | 7.36 | 7.34 | 0.61 | 1.97 | 1.36 | 2.34 |
| Fe_2_O_3_ | 0.16 | 0.42 | 0.73 | 1.27 | 0.17 | 0.53 | 0.97 | 1.84 | 0.057 | 0.29 | 1.24 | 1.87 |
| PbO | 0.00014 | 0.0013 | 0.049 | 0.07 | 0.00014 | 0.018 | 1.64 | 4.90 | NA | 0.038 | 5.48 | 9.49 |
| TiO_2_^*^ | 0.019 | 0.076 | 0.060 | 0.12 | 0.025 | 0.084 | 0.061 | 0.12 | 0.013 | 0.025 | 0.013 | 0.029 |
| MnO^*^ | 0.76 | 0.31 | 0.028 | 0.019 | 0.76 | 0.31 | 0.11 | 0.059 | NA | 0.25 | 0.13 | 0.12 |
| Ba^*^ | 32.05 | 48.26 | 201.36 | 111.29 | 37.42 | 48.54 | 201.09 | 110.39 | 11.77 | 20.41 | 33.23 | 27.85 |
| Zr^*^ | 8.05 | 88.64 | 36.35 | 115.90 | 15.41 | 113.94 | 39.38 | 125.68 | 12.29 | 56.60 | 9.82 | 49.30 |
| Sr^*^ | 168.26 | 266.11 | 448.65 | 220.69 | 167.49 | 253.11 | 442.22 | 205.60 | 5.92 | 60.13 | 83.21 | 45.61 |
| Nd^*^ | 0.79 | 4.01 | 6.08 | 6.36 | 1.12 | 3.98 | 6.15 | 6.44 | 0.61 | 0.58 | 0.75 | 1.18 |
| Th^*^ | 0.18 | 0.51 | 0.83 | 1.58 | 0.21 | 0.58 | 0.88 | 1.66 | 0.087 | 0.10 | 0.22 | 0.34 |
| La^*^ | 1.02 | 4.45 | 6.40 | 7.07 | 1.29 | 4.60 | 6.66 | 7.15 | 0.64 | 0.67 | 1.58 | 0.94 |


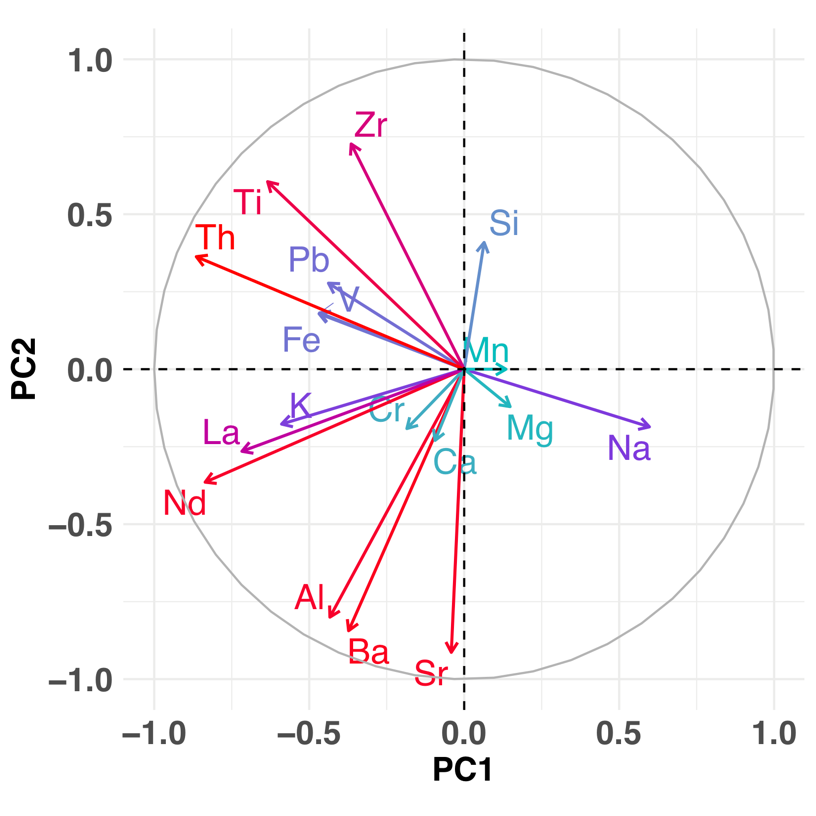


**Fig. S1. The contribution of the chemical elements under PCA examination**, shown by the quality of representations. The longer arrows that are closer to the circumference indicate the better represented elements in the principal components. Eight elements Ba, Zr, Ti, Sr, Nd, Th, Al and La are the best represented.


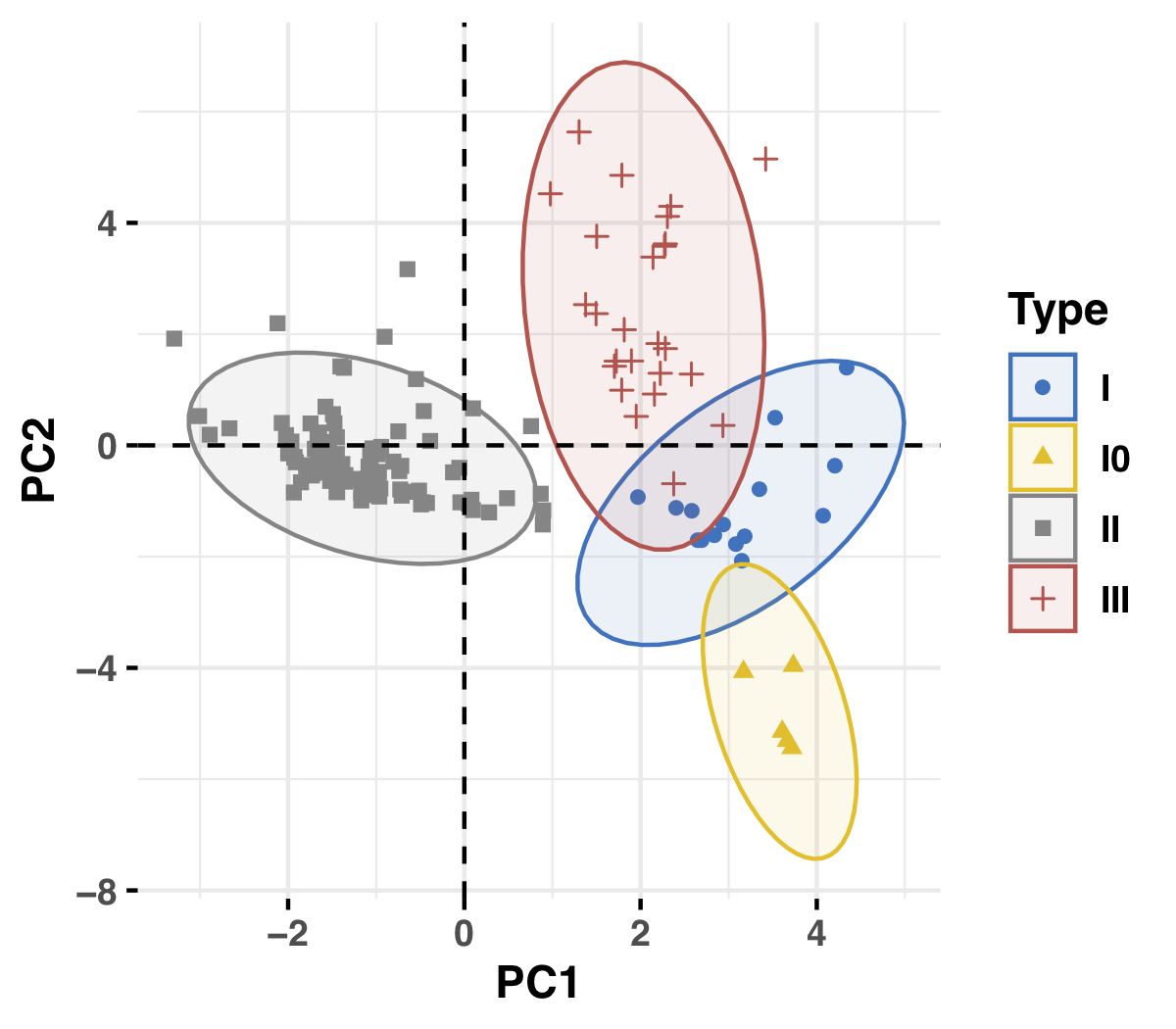


**Fig. S2. Four groups can be identified from the data points by PCA: I, II, III, and I_0_.** Each group (composition type) is marked by an ellipse of 95% confidence.

**The 1st millennium natron glass from the Mediterranean**

A large number of natron glass artifacts have been recovered from the Mediterranean and the Black Sea regions. Natron glass was discovered in a number of Iron Age sites in southern Italy (8-6 C. BCE) ^6,7^. The first natron glass on the island of Rhodes, Greece (late 7 C. BCE, the Archaic period) was likely imported ^2^. Chemical and strontium-neodymium isotopic compositions indicate that the raw glass used for most glasses from Methoni (8-4 C. BCE) and Pydna (6-4 C. BCE), Greece was possibly made on the Syro-Palestinian coast ^3^. A Syro-Palestinian origin was also suggested for the natron glass recovered in Dren-Delyan (6-4 C. BCE), Bulgaria ^5^. Natron glasses from Spina and Bologna, northern Italy (6-4 C. BCE) ^10^ and from Mozia, Sicily, southern Italy (6-4 C. BCE) ^11^ are relatively uniform in composition, for which a Greek origin was suggested. Rhodes was proposed as a center of glass production during the Classical and Hellenistic times ^12^. Natron glass from Pichvnari (5 C. BCE) on the Black Sea coast of Georgia may be associated with Syro-Palestinian production, and is similar to glass from Rhodes ^13^. A similar conclusion was reached for natron glass from Apollonia Pontica (5-3 C. BCE), Bulgaria ^14^. Among the natron-based core-formed glass vessels from Adria, northern Italy, the Mediterranean Group (MG) I (5 C. BCE), MG-II (3 C. BCE), and MG-III (2 C. BCE) were inferred to have different provenances. In particular, Adria's MG-I glass compositionally aligns with glass from Pichvnari and was suggested to originate from Rhodes ^4^. However, a study of elemental and isotopic compositions of MG-I vessels (6-4 C. BCE) ^15^ indicated that the Levant was still a more likely location for primary production of the glass for these vessels than Rhodes. An investigation of MG-II vessels (4-3 C. BCE) from Satricum, central-west Italy, suggested that they may be manufactured in multiple sources, including Italy and Rhodes ^1^. The raw glasses used to make the beads excavated from Son Mas, Mallorca, Spain were suggested to originate from the Levant (possibly 4-3 C. BCE) and Egypt (possibly 3 C. BCE), based on composition ^8^. Natron glass has also been found from Greek settlements of the Middle to Late Hellenistic contexts (3-1 C. BCE) ^16,17^.

In addition to these Mediterranean finds, natron glass was also discovered outside the Mediterranean coastal region in association with trade. Natron glasses have been reported from Germany (900-600 BCE ^18^ and 6-5 C. BCE ^19^), Poland (8-3 C. BCE ^20^ and 6-4 C. BCE ^19^), and as far as Mali (7-5 C. BCE) ^21^, all of which were suggested to originate from the Mediterranean.

**Additional plots for the compositional types**

Besides the bivariate plots in the main text (Fig. 1), other plots also show similar clusters of samples which can be attributed to the same four compositional types, affirming the consistency of our categorization. We show these additional plots in Fig. S3 and Fig. S4.

**Al versus Na (Fig. S3a).** Al is usually introduced by clay and feldspar that are often found in quartz sand. Among the types, Type II has the highest Al level, followed by Type III. Types I and I_0_ contain very little Al. Type I and Type I_0_ samples also contain the highest Na concentration on average. The average soda content in Type III glass is the lowest among the types. The variable range of soda implies that there could be more than one site of raw glass production for Type III.

**Sr versus Ca (Fig. S3b).** Sr atoms replace Ca atoms at a higher rate in shells and a lower rate in limestone. Types I, I_0_, and III have low Sr/CaO values, while Type II has high Sr/CaO values, confirming the sources suggested in the main text. This also implies that Types I, I_0_, and III may have similar original sources of Ca (limestone). Among Type II samples, as a general observation those from Italy mostly have higher Sr/CaO ratios than those from Greece or the Black Sea region. In Type III, some samples from Son Mas have high CaO levels and low Sr/CaO ratios.


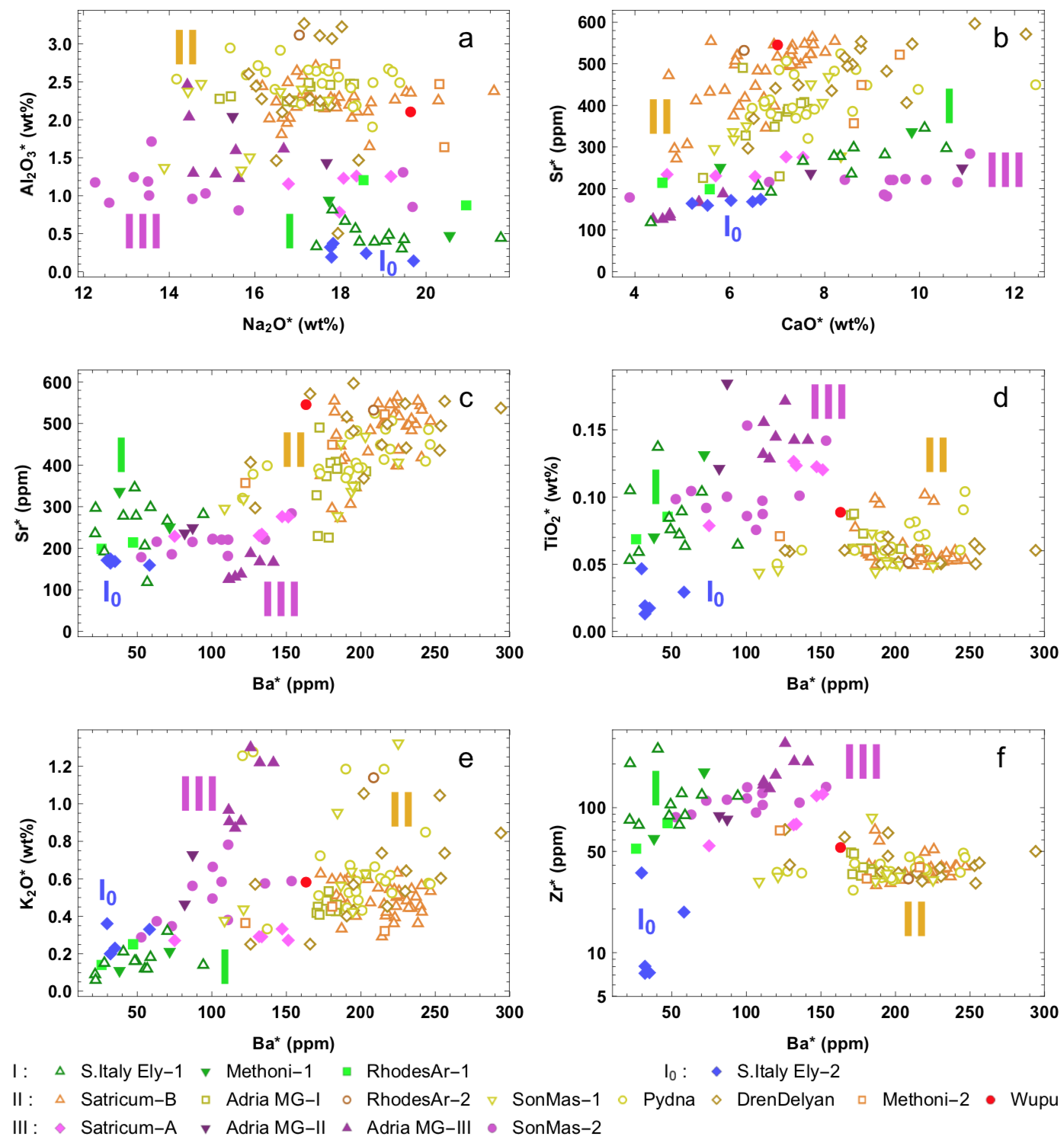


**Fig. S3. Additional bivariate plots of element concentrations for Mediterranean natron glass (8-2 C. BCE**). The figures show three compositional major types (I, II, and III) and one minor type (I_0_) for the samples. Refer to Fig. 1 in the main text for the description of the datasets.

**Sr, K versus Ba (Fig. S3c, e).** Barium could be introduced into glass in several ways. Ba can substitute for Sr and Ca atoms, and thus could come from shells or limestone in the vitrifying raw material. Ba can also be found in potassium feldspar and mica ^22^, which implies a relation with K or Rb. A previous study of Mediterranean beach sands reported a lack of correlation between Ba and K or Rb, and instead observed moderate and positive Ba-Sr and Ba-Ca correlations, raising the possibility of Ba existing in carbonates, albeit any shells analyzed reportedly contain little Ba ^23^. Alternatively, Ba can also exist in fractions deriving from sedimentary rocks mainly in the form of barite if it occurs ^22^, as was suggested for Roman glass from Hambach, Germany ^24^. A Ba-S correlation would attest to the presence of barite. In addition, manganese ores were also suggested as a possible source of Ba ^23^. Here we consider several possibilities for the sources of barium. As shown in Fig. S3c, both Ba and Sr are at high levels for Type II, which suggests that Ba is mainly introduced with Sr that comes with shells for Type II. The Sr levels of Types I, I_0_, and III are relatively low. Partial substitution of Sr and Ca by Ba likely took place, and is the main source of Ba for these types. For Type III, the Ba level is also contributed by another source: In Fig. S3e, a positive correlation exists between Ba and K for most Type III samples, indicating that potassium feldspar and mica likely provide Ba for these samples. No Ba-S or Ba-Mn dependencies were found, although sulfur data are often unavailable. The different origins of Ba for different compositional types reaffirm that distinct raw materials were used to make the raw glasses for these types.

**Ti, Zr versus Ba (Fig. S3d, f).** Since Zr and Ti are mostly related to heavy elements, no correlation is found between Zr or Ti and Ba, confirming they are from independent sources. The four groups are well discriminated in Figs. S3d and f.

**Zr versus Ti. Fig. S4a** shows overall positive correlations between Zr and TiO_2_ (expressed as their reciprocals). Type II artifacts are more clustered, while Types I and III artifacts are more spread out. Types I, I_0_, and III samples are distributed on somewhat similar slopes, while most Type II samples are on a less steep slope. The Ti levels in all these samples are generally low, with the lowest level found in Type I_0_. Most samples in Types I and II have TiO_2_^*^ ≤ 0.1%, while in Type III, TiO_2_^*^ < 0.2%. A low Ti level is the norm for most natron glasses in this period. In fact, natron glass was generally low in Ti until the 4th C. CE, after which more natron glasses with higher Ti emerged (for example ^25^ and data in ^26^).

**Th versus Nd, La (Fig. S4e, h).** In the Th-Nd relation (Fig. S4e), Type III has a very different trend compared to the other types. Fig. S4h displays the Th-La relation. La is a light REE mainly hosted in monazite and allanite. The positive correlations of Th-Nd and Th-La most likely derive from heavy minerals such as monazite that host both elements. In both plots, Type III samples are on a steeper slope than Types I_0_, I, and II, indicating different composition in heavy minerals, e.g., the sources for Types I_0_, I, and II may contain a higher percentage of monazite-(La).

**Nd, Sr vs Ti; Zr vs Nd (Fig. S4b, c, d).** These bivariate relations involve two elements from different geochemical element groups, thus displaying a clear separation of the four compositional types I, II, III and I_0_. Ti and Nd are loosely correlated except for Type II. The Zr-Nd relation is comparable to the Th-Zr relation (Fig. 1c) in the main text.

**Th vs Sr; La vs Zr (Fig. S4f, g).** The Th-Sr relation is comparable to the Nd-Sr plot in the main text (Fig. 1b). A positive and linear trend exists for Type II, which also extends to Type I. Type II is much richer in Sr than the rest, and has a relatively consistent level of Th. The La-Zr relation is comparable to the Th-Zr relation in Fig. 1c. Again, Type III's distinct heavy mineral profile is demonstrated in its high Th level and in its Th-Sr and La-Zr trends.


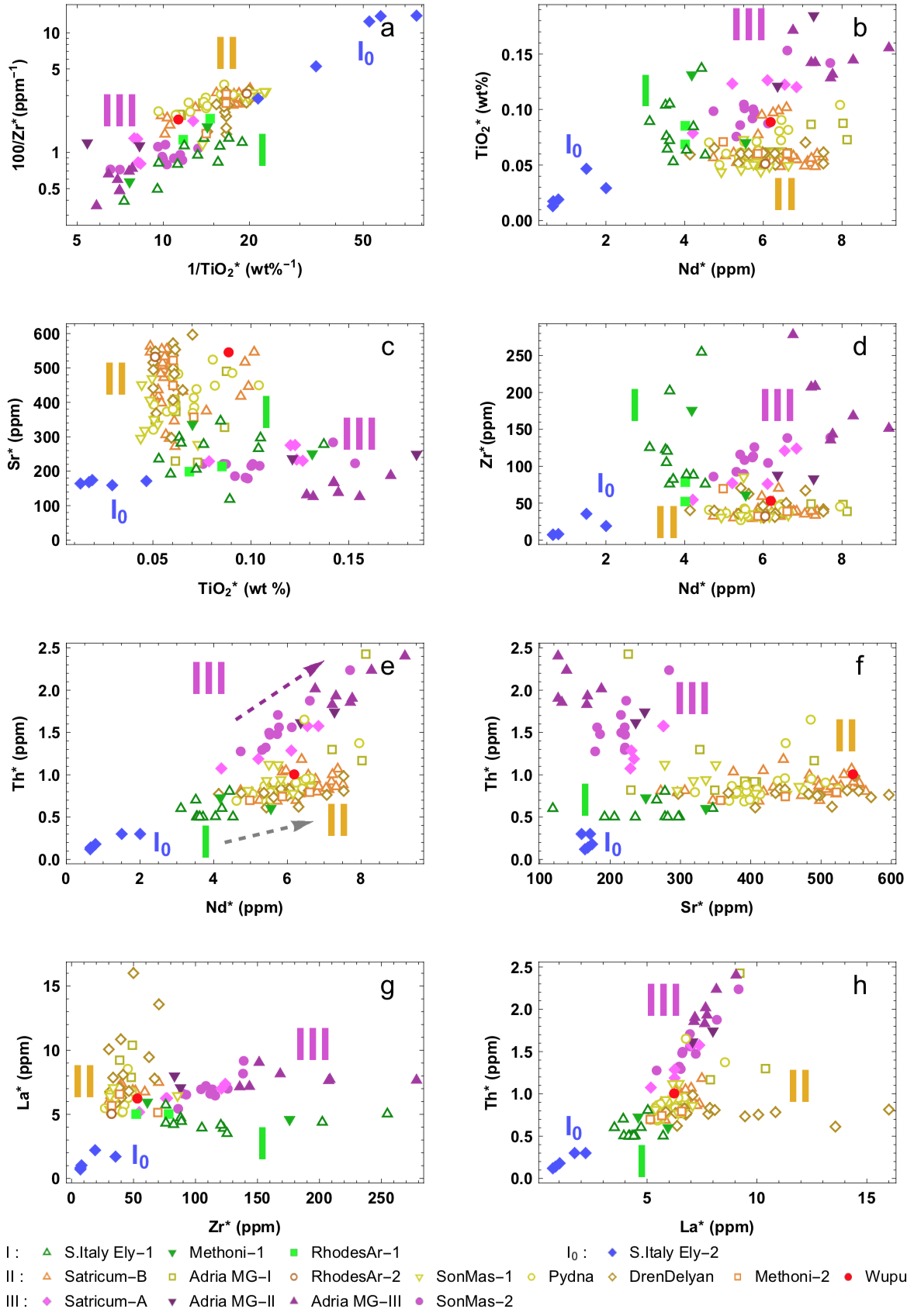


**Fig. S4. Additional bivariate plots of element concentrations for Mediterranean natron glass (8-2 C. BCE**), showing three compositional major types (I, II, and III) and one minor type (I_0_) for the samples. Refer to Fig. 1 in the main text for the description of the datasets.

**Compositional types of additional samples**

Additional assemblages of natron glass dated to the 8-2 C. BCE can also be attributed to the four compositional types. We note their attributions here but didn’t include them in the plots to avoid redundancy and to ensure figure readability. Natron glass found in Poland mostly belongs to Type I (Hallstatt C/D, 8-5 C. BCE) and Type II (Hallstatt D to Early La Tène, 7-3 C. BCE) ^20^. Natron glass samples from the Black Sea coastal sites including Pichvnari, Georgia (5 C. BCE) ^13^ and Apollonia Pontica, Bulgaria (5-3 C. BCE) ^14^, and from the middle to late Hellenistic sites in Greece including Demetrias (3-1 C. BCE) ^16^ and Epirus (3-1 C. BCE) ^17^, fit in Type II. Although without complete trace element data for a conclusive ascription, from the Al and Ti levels it is possible to speculate that natron glasses from Spina and Bologna, northern Italy ^10^, and Mozia, southern Italy ^11^ (6-4 C. BCE) belong to Type II.

**Sources of Type I_0_ and Type I**

In the main text, we proposed that Type I_0_ and Type I were probably produced in central Mediterranean. This is based on the geographical distribution of current glass finds of Types I_0_ and I, which may be updated in the future. On the other hand, we also note that the siliceous sources are increasingly sorted from Types I_0_ and I to Type II, which implies a shift of the location for mining raw materials from the piedmont towards the shore. The relatively short time range for this change may suggest a glass-making tradition’s adaption to resource availability within the same general region such as the east of the Mediterranean, considering the origin of Type II raw glass. Further archaeological evidence is required to validate any proposals for the raw material sources of early natron glass.

**Natron glass bead from Wupu**

**The sample.** The dimensions of the Wupu bead are 2.6 mm (height) by 3.4 mm by 3.4 mm, which is a symmetrical shape. The surface is mostly smooth although yellow surface corrosion is visible. The holes of the bead are slightly irregular, with the hole diameter measuring 0.8 ~ 0.9 mm. The two ends of the hole have similar diameters.

**The Wupu site's archaeological background.** Since 1978, 114 tombs have been excavated. Burial goods are mostly utilitarian, including woven clothing, leather shoes and hats, wooden baskets, bowls, and plates, as well as painted and non-painted pottery. Small bronze tools such as knives, bells, earrings, needles, and one bronze mirror were discovered, in addition to lithic tools and mammal bones ^27,28^. Exotic items such as seashells were also found. Human remains of both males and females, adults and children were recovered, and osteometric observation indicates the presence of both Mongolian and Caucasian groups ^29^. Beads found in Wupu include three globular blue glass beads of similar sizes, two globular black beads made of jet, one semi-transparent colorless bead of annular shape (material unknown), and many small, thin stone beads of various forms. Unfortunately, among glass beads, only one blue bead was allowed for us to analyze.

**Chronology.** The blue bead from Wupu was excavated in 1986. A date from specific stratigraphy is not available for this bead, therefore its chronology is delimited by the overall chronology of the site. Archaeological features such as burial goods indicate that the site was occupied from the Bronze Age to the Early Iron Age. No burials later than the Early Iron Age have been found. It was thus indicated that the excavated glass bead should have been buried before the late Spring and Autumn period. Recent efforts for dating the Wupu site have yielded consistent results. Four textile samples were sent for radiocarbon dating, with results given in Table S3. The results are comparable to previous dating results from plant remains^30^ and reaffirm the archaeological assessment. According to the dates obtained, the Wupu site belongs to a time range between the late 2nd millennium BCE and the first half of the 1st millennium BCE with two periods of settlement. By combining the dates for the latter settlement phase (78HWM14:3 and 86HWM70:15), we obtain a date of 760-481 BC (95.4%) for a 2σ uncertainty (Fig. S5). Corroborating this date, the combined date for the latter settlement phase using dates obtained with plant remains is 758-543 BC (95.4%).

**Table S3.** **Radiocarbon dates for textile samples from Wupu.** The radiocarbon dates listed as ^14^C BP (BP=before AD 1950) were obtained by Beta Analytic Testing Laboratory. We calibrated the radiocarbon dates into calendar years using OxCal 4.4 ^31^ and INTCAL20 curve ^32^, with time ranges expressed in cal.BC for 95.4% probability and 68.2% probability respectively.

| Sample No. | Description | Radiocarbon date 14C BP | Calibrated individual dates, cal.BC  (95.4% probability) | Calibrated individual dates, cal.BC  (68.2% probability) |
| --- | --- | --- | --- | --- |
| 86HWM53:9 | textile, trouser leg | 2820±30 BP | 1106BC ( 0.5%) 1098BC  1079BC ( 0.7%) 1069BC  1056BC (94.2%) 898BC | 1007BC (68.3%) 929BC |
| 86HWM55:8 | textile, wool clothing fragment | 2720±30 BP | 918BC (95.4%) 810BC | 898BC (68.3%) 827BC |
| 78HWM14:3 | textile, wool trousers | 2480±30 BP | 772BC (95.4%) 476BC | 756BC (14.5%) 718BC  708BC (11.2%) 680BC  670BC ( 3.3%) 661BC  654BC (18.0%) 606BC  596BC (21.3%) 543BC |
| 86HWM70:15 | textile, wool clothing fragment | 2460±30 BP | 758BC (29.3%) 678BC  671BC (60.5%) 452BC  446BC ( 5.7%) 416BC | 750BC (27.3%) 685BC  667BC (11.8%) 636BC  588BC ( 3.0%) 578BC  572BC (21.1%) 512BC  502BC ( 4.9%) 484BC |


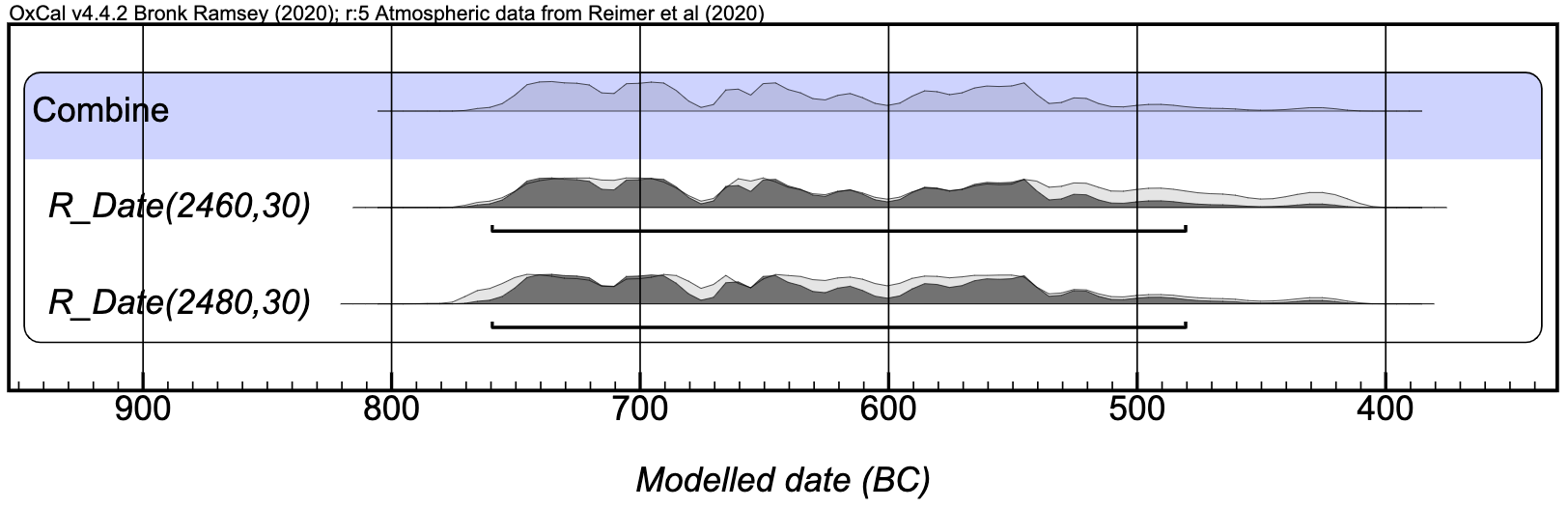


**Fig. S5. Wupu’s latter phase of settlement is determined by combining the dates from two textile samples.** The date obtained is 760-481 BC (95.4%) for a 2σ uncertainty.

**Early natron glass from China**

We provide information of potential natron glasses from China to complement the main text. More details can be found in the publications that originally reported these artifacts.

Natron glass normally has K and Mg contents lower than 1.5%. However, our judgment on whether the reported sample is natron-based was constrained by the extent of environmental corrosion and the reliability of the original analyses. Most of these beads were severely corroded, and some of them may not be well-vitrified, resulting in superficial and internal heterogeneity that create complications for chemical characterization. With corrosion present, surface layers are usually depleted in the alkali content (e.g. Na, K), and enriched in Si, Al, Fe, and possibly Ca, and abnormal corrosion can also occur ^33^. In addition, the quantitative reliability of analytical methods also greatly varies with different setups and with different chemical elements of interest. For example, an analysis (such as X-ray fluorescence) limited to the unprepared surface cannot render the pristine glass composition. Also, certain analytical methods (such as atmospheric analysis of Proton Induced X-ray Emission ^34^ or portable X-ray fluorescence) cannot obtain concentrations of light elements properly. Critically, in some cases, Na composition is either depleted or unreported. To identify potential natron glass, it is therefore necessary to carefully examine the available data. We adopted an empirical approach by making full use of the reported major elemental data. The reported levels of K and Mg were assessed based on how much Na, Ca, Si and other elements deviate from their typical ranges in soda glass. The more the deviations are, the less certain we can be about the original composition.

**Xujialing.** Eleven eye beads (HNZZ1-HNZZ11) were excavated from a tomb at Xujialing, Xichuan, southern Henan ^35^. The tomb belongs to a noble of the Chu state and is dated to the early Warring States. All beads have a blue body colored by Cu, and deep blue eye pupils colored by Co which are surrounded by multiple brown circles on white inlaid areas. These beads contain 5%-10% Na_2_O, 7%-15% CaO, 3%-5% Al_2_O_3_, less than 2.5% K_2_O, and less than 1.5% MgO, according to Proton Induced X-ray Emission (PIXE) analyses. In some samples, the post-corrosion K levels are higher than typical natron glass composition. According to X-ray diffraction (XRD) analysis, the bodies of these beads are vitrified but still contain particles, while the pupils and circles clearly contain crystals and are not glass in the full sense.

**Jiuxian.** The eye bead excavated from Jiuxian, Yexian, central Henan (HNWKII-83) is dated to the late Spring and Autumn period ^36^. The bead has a blue body, yellow pupils and white rings, and was analyzed with PIXE and XRF (X-ray fluorescence). Surface layers of the blue body were removed for portable XRF analysis. The results show that the blue body contains 15% Na_2_O, 1.3% MgO, 3.8% Al_2_O_3_, 0.4% K_2_O, 7% CaO, 2% CuO and some antimony. The white part contains high Sb (data don't add up to 100%). The authors also suggested two severely corroded glass beads dated to the Warring States from Luoyang, Henan Province as natron glass as well.

**Hougudui.** The eye bead from Hougudui Tomb I in Gushi, southern Henan (sample No. G2) is dated to the late Spring and Autumn period ^37^. It has a green body, and blue and white eyes. The green part is colored by Cu and is more transparent. The blue part is colored by Co. The white part is opaque and has particles trapped inside that contain As and Sb, with bubbles on the edge of the white glass. Only the EPMA data for a few elements in the green part were available: it contains 0.6% Fe_2_O_3_, 9.4% CaO, 0.4% MgO, 0.5% K_2_O, and 10.9% Na_2_O. XRD analysis shows all components are non-crystalline and considerably homogeneous. White and black sand grains are embedded on the surface in the hole.

**Zenghouyi.** A total of 173 eye beads were excavated from Zenghouyi Tomb, Suizhou, Hubei Province, dated to 433 BCE or slightly later. Three selections of these beads have been analyzed. XRF analysis of one bead (HB-6) ^38^ suggests its deep blue pupils, blue body and white-brown circles contain 79%-84% SiO_2_, about 8% Na_2_O (5% for the brown sample), 6% CaO, 0.2%-0.5% MgO, 0.6% K_2_O, 3% Al_2_O_3_, and small amounts of Sr. The two blue parts also contain about 1.3% Fe_2_O_3_. CuO is however at only 0.2% in all three parts. According to the PIXE data of two other eye beads from Zenghouyi (HBWKI-16, HBWKI-17) ^34^, we suggest they could be natron glass, but little Na is in the beads. The samples contain Cu, and the blue pupils are colored by Co and high in Fe (3%-5%). Finally, over 40 beads from Zenghouyi were analyzed by portable XRF and were found to be natron-based ^39^. The beads contain 1.69-9.26% NaO (mostly under 6.5%), 7-12% CaO, and some Sr. A few samples contain up to 9% PbO.

**Leigudun.** Situated in the same locale in Suizhou, Leigudun Tomb II is dated also to the Warring States but later than Zenghouyi. Wavelength dispersive XRF results ^40^ show that three eye beads (lgd1-lgd3) have compositions that are potentially of natron glass, containing 72-76% SiO_2_, 4%-5% Na_2_O, 0.3%-0.5% MgO, about 1% K_2_O, 2% Al_2_O_3_, 11% CaO, 1% SO_3_, indicating that the beads are mostly uniform in composition. The blue and brown parts contain 0.6%-1.6% Fe_2_O_3_, and the blue bodies contain around 1% CuO. The glass beads also contain 1%-5% Sb_2_O_3_ as the fining agent, with the white part having the highest Sb level. XRD analysis of lgd1 indicates the bead's body is well-vitrified whereas the eye parts still contain some quartz crystals. In another publication ^34^, an unspecified part in an eye bead (HBWKI-46) from Leigudun Tomb II was analyzed with PIXE, which shows it contains about 7.5% Na_2_O, 1.3% MgO, 0.2% P_2_O_5_, 1.2% K_2_O, 7% Al_2_O_3_ and 8% CaO.

**Majiayuan.** One eye bead (MJY-M4-25) excavated from Tomb M4, Majiayuan, Zhangjiachuan, Tianshui, Gansu Province in central-west China, dated to the late Warring States period, was analyzed with portable XRF ^41^. The bead consists of a white body, blue pupils, and brown rings, which contain 6.5%-9.5% Na_2_O, 67%-72% SiO_2_, 9.8%-10.8% CaO, and 5%-6% Sb_2_O_5_. Raman spectroscopy indicates calcium antimonate is used as an opacifier. The blue pupil contains 0.25% CoO, and is low in Mn and high in Fe.

**Additional Remarks**

**Black natron glass and Type III natron glass.** The normalized composition of early black natron glass from southern Italy (9-7 C. BCE) ^6,7,42^ is similar to Type III, except that its Ca, Sr, Ba, Zr contents are lower, and Fe, Ti levels are higher. Most of these compositional features are also seen in Egyptian black sand, which is rich in magnetite and ilmenite and abundant in REEs especially with the Nile Delta's placer deposit of monazite ^43^.

**Eye beads in the early classifications.** We identified in the main text four typologies of stratified eye beads that have been discovered both in the West and in China. Except fragment samples, all of the intact beads are round or lantern-like (slightly flattened on the ends) in shape. A few works from the early 20th century studied the forms of eye beads with information accessible at that time, which our investigation has *not* based on. The general forms of the beads in our discussion fall into I.C.1.a and I.C.2.b families in Beck's classification ^44^. At face value, the identified typologies appear like No. 53, 50/51, 40/42, and 54 in the hand-drawn illustrations by Eisen ^45^.

**Pokrovka glass beads.** Data for many trace elements are not available for natron glass beads from Pokrovka, Russia. Based on the Al and Ti contents, most of them may be made of Type II glass, although a definite attribution would need information from trace elements.

One black glass bead (high in Fe and Pb) was reported from Pokrovka, Russia. This bead was deemed as a Chinese product in the original publication ^46^. The authors stated that Ba was searched for but not detected. In fact, although lead-barium glass is a Chinese invention in the 1st millennium BCE, the lead content was almost always accompanied by Ba, and the high-Pb glass without Ba was only developed much later in China. On the other hand, high-Fe, high-Pb glass has been found in the Mediterranean (e.g. Italy ^7^). Therefore, since other information about the bead is not available to us, we tentatively suggest this bead has a Mediterranean origin and is possibly an early product.

1. Oikonomou, A., Henderson, J., Gnade, M., Chenery, S. & Zacharias, N. An archaeometric study of Hellenistic glass vessels: evidence for multiple sources. *Archaeol. Anthropol. Sci.* **10**, 97–110 (2018).

2. Oikonomou, A. & Triantafyllidis, P. An archaeometric study of Archaic glass from Rhodes, Greece: Technological and provenance issues. *J. Archaeol. Sci. Reports* **22**, 493–505 (2018).

3. Blomme, A. *et al.* Provenance of polychrome and colourless 8th-4th century BC glass from Pieria, Greece: a chemical and isotopic approach. *J. Archaeol. Sci.* **78**, 134–146 (2017).

4. Panighello, S., Orsega, E. F., van Elteren, J. T. & Šelih, V. S. Analysis of polychrome Iron Age glass vessels from Mediterranean I, II and III groups by LA-ICP-MS. *J. Archaeol. Sci.* **39**, 2945–2955 (2012).

5. Tzankova, N. & Mihaylov, P. Chemical characterization of glass beads from the necropolis of Dren-Delyan (6th-4th century BC), Southwest Bulgaria. *Geol. Balc.* **48**, 31–50 (2019).

6. Conte, S. *et al.* How much is known about glassy materials in Bronze and Iron Age Italy? New data and general overview. *Archaeol. Anthropol. Sci.* **11**, 1813–1841 (2019).

7. Conte, S., Arletti, R., Mermati, F. & Gratuze, B. Unravelling the Iron Age glass trade in southern Italy: the first trace-element analyses. *Eur. J. Mineral.* **28**, 409–433 (2016).

8. Van Strydonck, M., Gratuze, B., Rolland, J. & De Mulder, G. An archaeometric study of some pre-Roman glass beads from Son Mas (Mallorca, Spain). *J. Archaeol. Sci. Reports* **17**, 491–499 (2018).

9. Degryse, P. Glass making in the Greco-Roman world: results of the ARCHGLASS project. in *Studies in Archaeological Sciences* vol. 4 189 (Leuven University Press, 2014).

10. Arletti, R., Maiorano, C., Ferrari, D., Vezzalini, G. & Quartieri, S. The first archaeometric data on polychrome Iron Age glass from sites located in northern Italy. *J. Archaeol. Sci.* **37**, 703–712 (2010).

11. Arletti, R., Ferrari, D. & Vezzalini, G. Pre-Roman glass from Mozia (Sicily-Italy): the first archaeometrical data. *J. Archaeol. Sci.* **39**, 3396–3401 (2012).

12. Triantafyllidis, P. New evidence of the glass manufacture in Classical and Hellenistic Rhodes. in *Annales du 14e Congres de l’Association Internationale pour l’Histoire du Verre - 1998* 30–34 (2000).

13. Shortland, A. J. & Schroeder, H. Analysis of first millennium BC glass vessels and beads from the Pichvnari necropolis, Georgia. *Archaeometry* **51**, 947–965 (2009).

14. Lyubomirova, V., Šmit, Ž., Fajfar, H. & Kuleff, I. Chemical composition of glass beads from the necropolis of Apollonia Pontifica (5th-3rd century BC). *Archaeol. Bulg.* **18**, 1–17 (2014).

15. Blomme, A. *et al.* Tracing the primary production location of core-formed glass vessels, Mediterranean Group I. *J. Archaeol. Sci. Reports* **5**, 1–9 (2016).

16. Smirniou, M., Gratuze, B., Asderaki, E. & Nikolaou, E. Chemical compositional analysis of glass from the north cemetery of ancient Demetrias (Thessaly). *J. Archaeol. Sci. Reports* **22**, 506–512 (2018).

17. Oikonomou, A. Hellenistic core formed glass from Epirus, Greece. A technological and provenance study. *J. Archaeol. Sci. Reports* **22**, 513–523 (2018).

18. Hartmann, G., Kappel, I., Grote, K. & Arndt, B. Chemistry and technology of prehistoric glass from Lower Saxony and Hesse. *J. Archaeol. Sci.* **24**, 547–559 (1997).

19. Agua, F. *et al.* Chemical-physical characterisation of Early Iron Age glass beads from Central Europe. *Boletín la Soc. Española Cerámica y Vidr.* **56**, 119–130 (2017).

20. Purowski, T., Syta, O. & Wagner, B. Between east and west: Glass beads from the eighth to third centuries bce from Poland. *Archaeometry* **62**, 752–773 (2020).

21. Giachet, M. T., Gratuze, B., Ozainne, S., Mayor, A. & Huysecom, E. A Phoenician glass eye bead from 7th-5th c. cal BCE Nin-Bèrè 3, Mali: Compositional characterisation by LA-ICP-MS. *J. Archaeol. Sci. Reports* **24**, 748–758 (2019).

22. Johnson, C. A., Piatak, N. M. & Miller, M. M. Chapter D: Barite (Barium). in *Critical Mineral Resources of the United States: Economic and Environmental Geology and Prospects for Future Supply* (U.S. Geological Survey, 2018).

23. Brems, D. & Degryse, P. Trace element analysis in provenancing Roman glass-making. *Archaeometry* **56**, 116–136 (2014).

24. Wedepohl, K. H., Simon, K. & Kronz, A. Data on 61 chemical elements for the characterization of three major glass compositions in Late Antiquity and the Middle Ages. *Archaeometry* **53**, 81–102 (2011).

25. Aerts, A., Velde, B., Janssens, K. & Dijkman, W. Change in silica sources in Roman and post-Roman glass. *Spectrochim. Acta Part B At. Spectrosc.* **58**, 659–667 (2003).

26. Brill, R. H. *Chemical Analyses of Early Glasses: Volume 2: Tables of Analyses*. (Corning Museum of Glass, 1999).

27. Wang, B. H., Liu, J. L., Mei, Y. X., Zhang, Y. X. & Lü, X. X. A study on the barley excavated from the Wupu burials, Hami, Xinjiang. *Agric. Archaeol.* **1**, 70 (1989).

28. Mei, J., Liu, G. & Chang, X. Preliminary analysis and research on early bronze excavated from Eastern Xinjiang. *West. Reg. Stud.* **2**, 1–10 (2002).

29. He, H. Q. & Xu, Y. Q. Anthropological study of ancient human skulls from Wupu site, Hami, Xinjiang. *Acta Anthropol. Sin.* **2**, 102–110 (2002).

30. Schröder, O. *et al.* Ancient DNA identification of domestic animals used for leather objects in Central Asia during the Bronze Age. *The Holocene* **26**, 1722–1729 (2016).

31. Ramsey, C. B. Bayesian analysis of radiocarbon dates. *Radiocarbon* **51**, 337–360 (2009).

32. Reimer, P. J. *et al.* The IntCal20 Northern Hemisphere Radiocarbon Age Calibration Curve (0-55 cal kBP). *Radiocarbon* **62**, 725–757 (2020).

33. Lü, Q.-Q. & Wu, Y. LA-ICP-MS analysis of corroded glass beads from Southern China: tackling highly inhomogeneous archaeological glass. *STAR Sci. Technol. Archaeol. Res.* **5**, 53–63 (2019).

34. Gan, F., Zhao, H., Li, Q., Li, L. & Cheng, H. Technological analyses on ancient glass of the Warring States period in Hubei province. *Jianghan Archaeol.* **2**, 108–116 (2010).

35. Gan, F., Cheng, H., Hu, Y., Ma, B. & Gu, D. Study on the most early glass eye-beads in China unearthed from Xu Jialing Tomb in Xichuan of Henan Province, China. *Sci. China Ser. E Technol. Sci.* **52**, 922–927 (2009).

36. Dong, J. *et al.* Non-destructive analysis of some glass artifacts dated from Eastern Zhou to Song Dynasty unearthed from Henan Province. *Mater. China* **31**, 9–15 (2012).

37. Zhang, F., Cheng, Z. & Zhang, Z. Research on ancient Chinese Liuli. *J. Chinese Ceram. Soc.* **1**, 67–76 (1983).

38. Li, Q., Zhou, H., Huang, J., Gan, F. & Zhang, P. Chemical composition analytic results of ancient Chinese compound eye-beads. *Jianghan Archaeol.* **4**, 79–86 (2005).

39. Zhao, H. X., Li, Q. H., Liu, S., Li, L. & Gan, F. X. In situ analysis of stratified glass eye beads from the tomb of Marquis Yi of the Zeng State in Hubei Province, China using XRF and micro-Raman spectrometry. *X-Ray Spectrom.* **43**, 316–324 (2014).

40. Qin, Y., She, L., Li, X. & Huang, J. Composition and structure of Warring States glasses from tomb two at the Leigudun site of Suizou, Hubei, China. *J. Chinese Ceram. Soc.* **37**, 574–576 (2009).

41. Huang, X., Yan, J. & Wang, H. Analysis of the Decorated Silicate Beads Excavated from Tomb M4 of the Ma-Jia-Yuan Warring States Cemetery, Gansu Province. *Spectrosc. Spectr. Anal.* **35**, 2895–2900 (2015).

42. Conte, S., Arletti, R., Henderson, J., Degryse, P. & Blomme, A. Different glassmaking technologies in the production of Iron Age black glass from Italy and Slovakia. *Archaeol. Anthropol. Sci.* **10**, 503–521 (2018).

43. Jackson, W. D. & Christiansen, G. International strategic minerals inventory summary report - rare-earth oxides. in *USGS Numbered Series: Circular 930-N* (U.S. Geological Survey, 1993).

44. Beck, H. C. I.—Classification and nomenclature of beads and pendants. *Archaeologia* **77**, 1–76 (1928).

45. Eisen, G. The characteristics of eye beads from the earliest times to the present. *Am. J. Archaeol.* **20**, 1–27 (1916).

46. Hall, M. E. & Yablonsky, L. Chemical analyses of Sarmatian glass beads from Pokrovka, Russia. *J. Archaeol. Sci.* **25**, 1239–1245 (1998).
